# The Complex Exogenous RNA Spectra in Human Plasma: An Interface with Human Gut Biota?

**DOI:** 10.1371/journal.pone.0051009

**Published:** 2012-12-10

**Authors:** Kai Wang, Hong Li, Yue Yuan, Alton Etheridge, Yong Zhou, David Huang, Paul Wilmes, David Galas

**Affiliations:** 1 Institute for Systems Biology, Seattle, Washington, United States of America; 2 Luxembourg Center for Systems Biomedicine, University of Luxembourg, Luxembourg City, Luxembourg; 3 Pacific Northwest Diabetes Research, Seattle, Washington, United States of America; Cardiovascular Research Institute Maastricht, Maastricht University, The Netherlands

## Abstract

Human plasma has long been a rich source for biomarker discovery. It has recently become clear that plasma RNA molecules, such as microRNA, in addition to proteins are common and can serve as biomarkers. Surveying human plasma for microRNA biomarkers using next generation sequencing technology, we observed that a significant fraction of the circulating RNA appear to originate from exogenous species. With careful analysis of sequence error statistics and other controls, we demonstrated that there is a wide range of RNA from many different organisms, including bacteria and fungi as well as from other species. These RNAs may be associated with protein, lipid or other molecules protecting them from RNase activity in plasma. Some of these RNAs are detected in intracellular complexes and may be able to influence cellular activities under *in* *vitro* conditions. These findings raise the possibility that plasma RNAs of exogenous origin may serve as signaling molecules mediating for example the human-microbiome interaction and may affect and/or indicate the state of human health.

## Introduction

Many novel biological insights have emerged from the analysis of DNA and RNA sequences. Important discoveries, such as various pathology-causing variants in the human genome and the history of human migration, were made possible by the availability of sequencing technology [1], [2], [3]. Normal human physiology is the result of a well-orchestrated balance between genetic (intrinsic) and environmental (extrinsic) factors, and the availability of the complete human genome sequence facilitates the study of complex human-environmental interactions. Recently this has included the human-microbiome interaction, especially the gut microbiome [4]. These microbes interact intimately with gut epithelium and the alteration in the spectrum of the gut microbiome has been linked to various physiopathological conditions, such as diarrhea, obesity, and inflammatory pathologies as well as to the general state of health [5], [6].

The recent development of highly parallelized next generation (NextGen) sequencing technologies has further advanced the use of sequencing as a tool in studying complex biological systems by genome sequencing and transcriptome analysis [7], [8], [9], [10]. One advantage of using a sequence-based approach for transcriptome analysis is the ability to identify novel transcripts, such as alternative usage of exons or polyadenylation sites of known transcripts. The recent explosion of information on microRNA (miRNA) and other noncoding RNAs (ncRNAs) is the result in part of applying these new technologies. MiRNAs are transcribed from genome by processes similar to protein-coding genes. The primary miRNA transcripts are processed in the nucleus and later in the cytosol by the RNase III enzymes Drosha and Dicer, respectively [11]. Typically, one strand of this mature miRNA duplex then associates with the RNA-induced silencing complex (RISC) where it interacts with its messenger RNA (mRNA) targets. To date more than 1000 different human miRNA species have been identified (see miRBase, www.mirbase.org). Recently, a significant number of these RNA molecules have been observed in the extracellular environment and have been implicated as important mediators in cell-cell communication [4], [12], [13].

## Results

### Low Fraction of Mappable Sequence Reads from Plasma Samples

Because of the shortcomings of existing miRNA measuring systems, we adapted the NextGen sequencing technology to obtain more accurate spectra of these important molecules in circulation, specifically to explore the plasma-miRNA association with colorectal cancer and ulcerative colitis. Initially we conducted NextGen sequencing on 9 plasma samples: 3 samples from healthy individuals, 3 from patients with colorectal cancer prior to any treatment and 3 from individuals suffering from ulcerative colitis (Mayo Score between 10 and 11) (Table S1). Sequence reads were preprocessed and then aligned to known human miRNAs, human transcripts and human genome sequence. The concentration of several miRNAs in plasma showed differences among normal and patients with either colorectal cancer or ulcerative colitis. We conducted quantitative polymerase chain reaction (QPCR) measurements to validate some of these miRNAs (Figures S1a and S1b).

On first examination, we noticed that less than 1.5% of the processed reads actually mapped to human miRNAs. About 11% of the remaining reads mapped to human transcripts and human genome sequence when no sequence mismatch was allowed (Table S3). With a higher tolerance of sequence mismatches, the fraction of reads that can be mapped to known human transcripts rose to about 42% and 15% to other human genomic sequences (under two mismatch allowance). However, this still leaves over 40% of the processed reads with an unknown origin.

To ensure our protocol is effectively mapping back the reads to transcript and genome sequences, a NextGen sequence read simulator, ART [14] (http://bioinformatics.joyhz.com/ART/), was used to generate artificial transcriptome data. With a 2 mismatch allowance, over 98% of the sequences from our simulated dataset can be mapped to the corresponding transcriptome (Table S4). This provided some assurance that our protocol can map most (∼98%) of the NextGen sequencing data under 2 mismatch allowance.

### Human Plasma Contains a Significant Amount of Exogenous RNA

**Figure 1:**
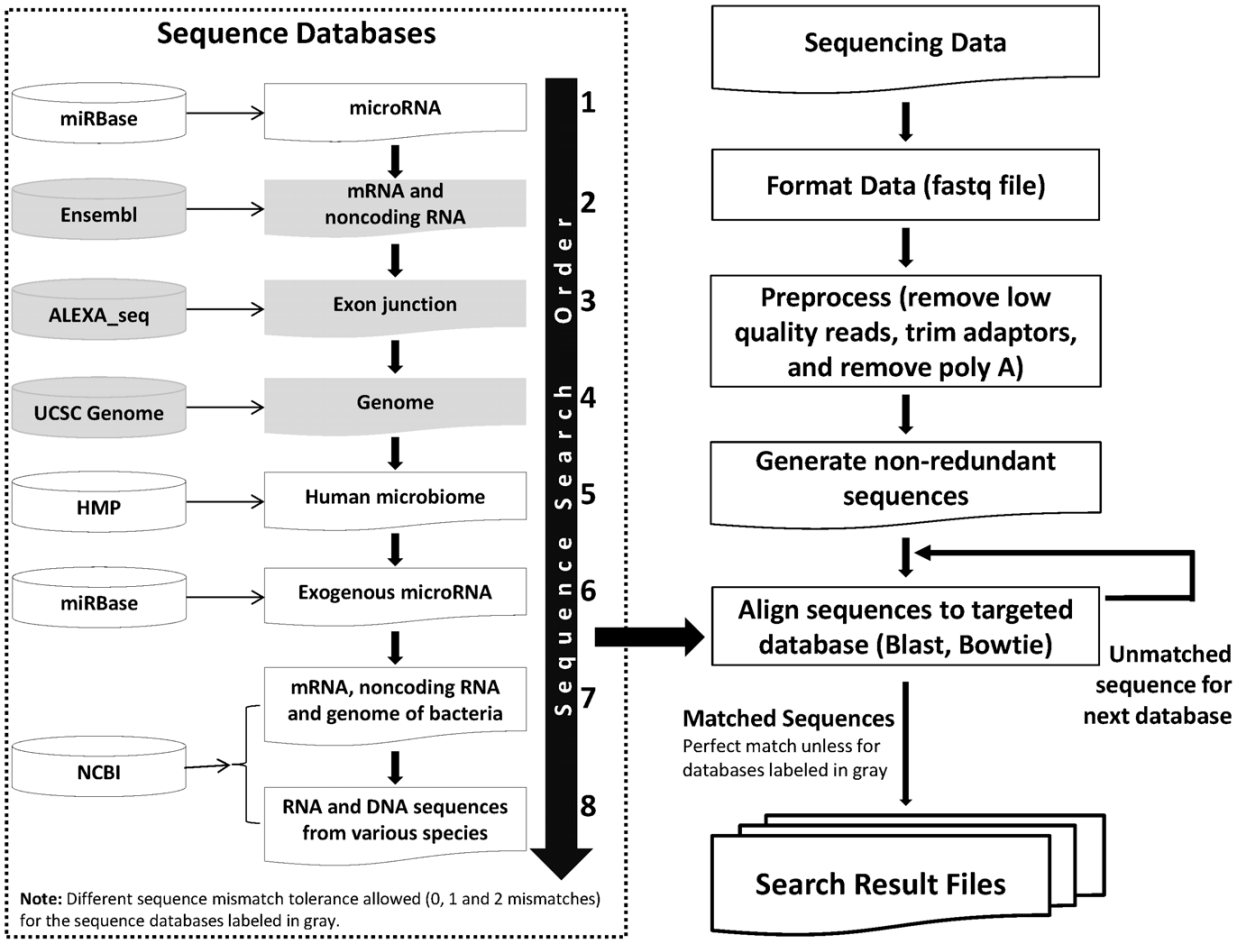
The schema of the sequence mapping protocol. A “map and remove” process was adapted to map reads against various sequence databases (left dotted box) in specific order as indicated. We allowed different levels of sequence mismatch tolerance, 0 mismatch, 1 mismatch and 2 mismatches when comparing the sequence reads against human sequence database.

**Figure 2:**
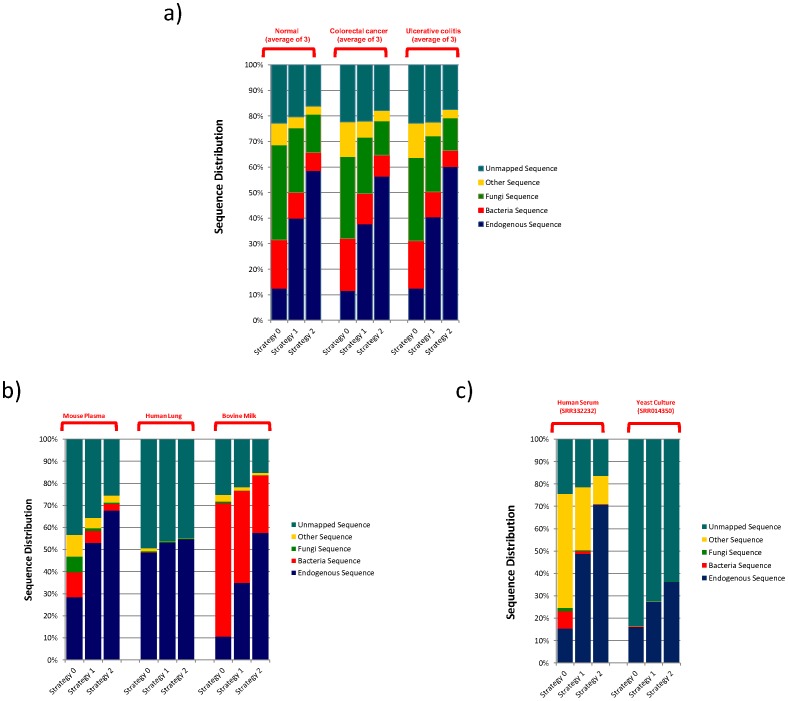
Distribution of sequence reads from human plasma (A), other sample types (B) and public domain data (C) among different sequence categories. The sample identifies were listed on the top, the sequence mapping criteria were indicated on the bottom and the list of different sequence categories is indicated on the right of each figure. See text for detailed description of Strategy 0, Strategy 1 and Strategy 2 sequence mapping criteria.

In order to identify the origin of those unmapped sequences in our sequencing results and to ensure that there was no error introduced in preparing the sequencing library that could account for the unknowns, we conducted a systematic search against various sequence databases. We used a “map and remove” approach to analyze the sequence (Figure 1). The processed sequences were first screened against endogenous (human) sequence databases including known human miRNA, human transcripts, followed by human genomic sequence. Except for the miRNA (since some of the miRNAs have very similar sequences), we applied three different levels of error tolerance, 0 mismatch (termed Strategy 0), 1 mismatch (termed Strategy 1) and 2 mismatches (termed Strategy 2) for the endogenous sequence mapping. The remaining unmapped sequences were then compared to sequences from the known human microbiome, miRNA sequences from other species, and the non-redundant nucleic acid sequence collection from NCBI without any mismatch allowance. To our surprise, a significant number of the unmapped reads aligned with various bacterial and fungal sequences (Figure 2a and Table S5).

### Exogenous RNA is also Observed in Other Sample Types

To eliminate the possibility of bacteria and fungi contamination during plasma preparation and handling, we generated sequencing libraries from other types of samples including human tissue (commercially obtained normal lung RNA), bovine milk (commercial whole milk), and mouse plasma (C57BL/6J), and proceeded through the same analysis scheme. Sequences from bacteria, fungi and other species can also be seen in these samples (Figure 2b and Table S6). The overall percentages of exogenous sequences for mouse plasma were lower compared to human plasma samples. The human lung tissue had a very small fraction: less than 1% under strategies 1 and 2, of the processed sequences were from exogenous sources. The commercially obtained milk contains a significant fraction of sequences attributable to bacteria.

To ensure that the exogenous sequences we observed were not derived from any contaminated instruments or reagents, we analyzed two public domain NextGen sequencing data sets: SRR332232, serum small RNA sequencing results from a normal Chinese individual [15], and SRR014350, yeast transcriptome data from a yeast culture [16]. The yeast culture should not have any exogenous sequences since it was grown in a sterile, defined culture media. The yeast dataset yielded less than 0.15% of the reads mapped to sequences other than yeast (Figure 2c and Table S7), a level that is fully attributable to coincidence caused by sequencing errors. Using our sequencing analysis pipeline, by contrast, we observed that about 12% of the sequences in human serum sample were from various exogenous species under Strategy 2.

To exclude the possibility that the observed exogenous RNAs were from intact bacteria and fungi contamination in our plasma samples, we used the 0.2 uM filter commonly used in tissue culture to eliminate bacteria and fungi contamination, to filter the plasma samples before RNA isolation. We did not observe any significant difference in exogenous RNA levels between filtered and unfiltered plasma, using QPCR primers specific to *Pseudomonas putida* 16S RNA and *Ceratocystiopsis minuta* 18S RNA, matching the results for the human 28S rRNA (Figure S2).

### Human Plasma Contains Sequences that Map to a Diverse Group of Microbiome Species

Based on the results from simulated dataset, allowing 2 mismatches should identify more than 98% of the endogenous sequences (Table S4). Therefore, the exogenous sequence mapping results from Strategy 2 (2 mismatches allowed for endogenous sequence mapping steps and no mismatch allowed in exogenous sequence mapping) were used for further analysis.

#### Bacteria and archaea

**Figure 3:**
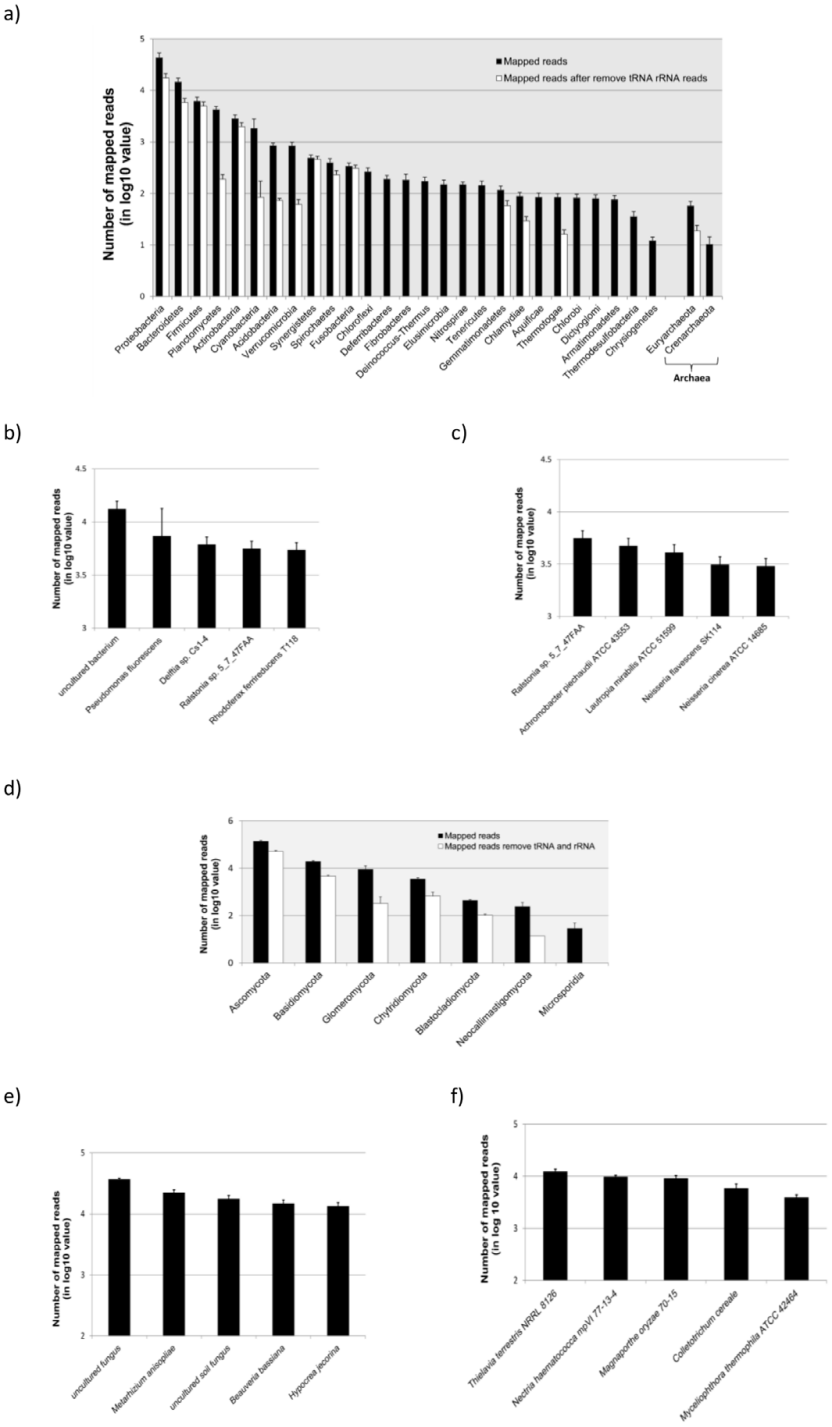
Distribution of sequence reads from human plasma mapped to bacteria, archaea (A to C) and fungi (D to F) phylum. The Y-axes are the numbers of reads in log 10 value and individual phylum are indicated on the X-axis. The number of reads used in the figures represents the average of all 9 plasma samples used in the study. The solid bars represent the total number of processed reads mapped to specific phyla while open bars are the number after removing rRNA and tRNA reads. The individual bacteria and fungi species with the most abundant processed reads (B and E) and processed reads after removing tRNA and rRNAs (C and F) are also shown.

We observed reads from plasma covering all major bacteria phyla and two archaea phyla (*Euryarchaeota* and *Crenarchaeota*) (Figure 3a and Table S8). We did not observe any significant difference in the sequence distribution patterns among plasma samples from normals and patients with either colorectal cancer or ulcerative colitis (Table S8). *Firmicutes*, typically the most abundant bacteria phylum in the human gut microbiome [4], is the 3^rd^ most abundant sequence population in plasma.

A significant number of the reads mapped to bacteria are from various ribosomal RNAs and tRNAs. High sequence similarity of these sequences among different microbial species can lead to misassignment of sequence reads. Thus, to increase the reliability of mapping results, we removed reads that mapped to bacterial rRNAs and tRNAs and reanalyzed the remaining reads. Removing rRNA and tRNA sequences affected our ability to detect species from *Chloroflexi*, *Deferribacteres*, *Fibrobacteres* and other phyla (Figure 3a open bars and Table S8). However, the *Proteobacteria* was still the most abundant phylum followed by *Bacteroidetes* and *Firmicutes*.

The bacterium that accounts for the highest number of reads is an uncultured bacterium. This is followed by *Pseudomonas fluorescens,* an important beneficial bacterium in agricultural settings [17] (Figure 3b). After removing the tRNA and rRNA reads, bacteria from the genus *Ralstonia* spp. become the most abundant source followed by *Achromobascter piechaudii*, a bacterium identified in some clinical blood samples (Figure 3c) [18].

#### Fungi

Fungi represent the largest source of exogenous RNA, about 14% of the processed reads under the Strategy 2 in our plasma samples (Table S5). Like bacteria, the species mapped covered all major fungal phyla, and *Ascomycota* is the most abundant phylum either with or without considering rRNA and tRNA reads (Figure 3d and Table S8). We could not detect species from *Microsporidia* following the removal of rRNA and tRNA sequences.

*Metarhizium anisopliae*, a common fungus in soil had the most mapped reads and *Thielavia terrestris,* a thermophilic fungus became the species with the most abundant reads after removing tRNA and rRNA sequences (Figure 3e and 3f). Either with or without rRNAs and tRNAs reads, we observed a significant number of reads mapped to yeast (*Saccharomyces cerevisiae*) which is commonly used in baking and brewing (Figure S3).

### Plasma also Contains Exogenous RNA Sequences from Common Foods

**Figure 4:**
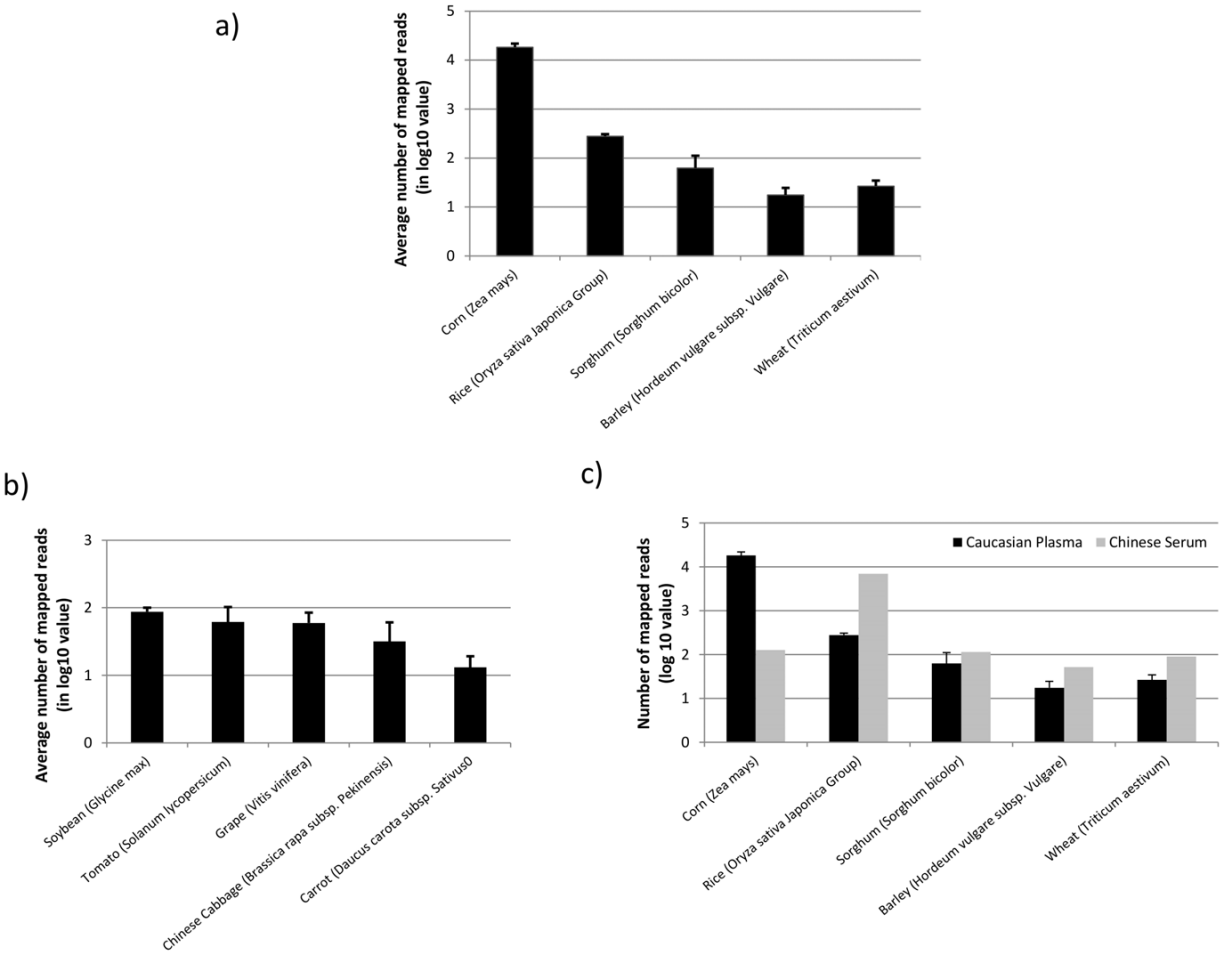
Number of sequence reads mapped to common food items such as cereal grains (A) and others (B). The Y-axes are the number of reads in log 10 value and individual species are indicated on the X-axis. The number of reads used in the figures represented the averages from all 9 plasma samples used in the study. Figure 4C shows the difference in the abundance of reads mapped to common cereal gains between serum sample from a Chinese individual (open bars) and the plasma samples (Caucasian) used in the study (solid bars).

After carefully examining the sequences mapped to species other than bacteria and fungi, we observed a significant number of processed reads that mapped to common food items. As we did for bacteria and fungi, we removed all the reads that mapped to rRNAs and tRNAs to increase the accuracy of mapping results. We did not analyze sequences mapped to metazoan species since the risk of coincidental sequence match caused by sequencing error is much higher between human and other metazoan samples. The most abundant food item derived RNA sequences identified from our plasma samples then are corn (*Zea mays*) followed by rice (*Oryza sativa Japonica Group*) (Figure 4a). The number of mapped reads to corn is 66 times higher on average than rice. In comparing the data from a serum sample from a Chinese individual (downloaded from the public domain: SRR332232), we found that the sequence abundance between corn and rice is reversed: rice has the highest number of reads, about 55-fold times the number from corn (Figure 4b). Besides the common cereal grains, we also observed RNA from other food items including soybeans (*Glycine max*), tomato (*Solanum lycopersicum*), grape (*Vitis vinifera*) and others in our plasma samples (Figure 4c).

### Plasma Contains Exogenous miRNAs from Other Species

Our sequencing results also revealed the presence of exogenous miRNAs from other species. Due to the extreme sequence similarity of miRNA sequences among some species, it is often difficult to determine the exact origin of those exogenous miRNAs. Some of the highly abundant exogenous miRNA species detected in our plasma samples are listed in Table 1. Except for miR-168a from the common cereal grains such as corn or rice, the rest of the exogenous miRNAs were from various common household insects, including the housefly, mosquito and bees. One interesting observation is the high variation in the number of reads among individual donors for those insect miRNAs. This was probably caused by the different living conditions and levels of contaminated food consumed by our donors, but this remains to be investigated.

**Table 1:** List of some abundant exogenous miRNA species identified in plasma.

Sample ID	Normal			Colorectal cancer			Ulcerative colitis		
	D3340P	D3176P	D3142P	022273P	022163P	022299P	93163P	93164P	93166P
**tca-miR-263a-5p**	678	3	2	1	1	0	57	2	6
**nvi-bantam; tca-bantam; dpu-bantam; isc-bantam; ame-bantam**	0	0	0	2	1	0	173	3	1
**zma-miR168a; sbi-miR168; sof-miR168a; osa-miR168a; ssp-miR168a; bdi-miR168; hvu-miR168-5p; zma-miR168b**	12	13	10	7	6	16	6	8	9
**dan-bantam; dwi-bantam; dme-bantam; dps-bantam; dgr-bantam;** **dya-bantam; aae-bantam; dse-bantam; dmo-bantam; dvi-bantam;** **dsi-bantam; der-bantam; dpe-bantam**	52	1	1	0	0	0	0	0	2
**dps-miR-8; ame-miR-8; dgr-miR-8; dme-miR-8-3p; cte-miR-8; nvi-miR-8;** **dwi-miR-8; isc-miR-8; tca-miR-8-3p; dpe-miR-8; nlo-miR-8; der-miR-8;** **dan-miR-8; lgi-miR-8; bmo-miR-8; aae-miR-8; aga-miR-8; dya-miR-8;** **dse-miR-8; dvi-miR-8; dsi-miR-8; dpu-miR-8; dmo-miR-8**	22	0	1	0	0	0	0	1	2
**bma-miR-228**	0	0	8	0	0	0	0	0	0
**cte-miR-252a; dsi-miR-252; dps-miR-252; sko-miR-252a; nvi-miR-252;** **dme-miR-252-5p; cqu-miR-252; bmo-miR-252**	14	0	0	0	0	0	2	0	0
**api-miR-263b**	0	0	0	0	0	0	10	0	0
**dpu-mir-263a; aae-mir-263a; cqu-mir-263; bmo-mir-263a**	9	0	0	0	0	0	1	0	1

### The Exogenous RNA may form Complexes with Proteins and Lipid in Plasma

It has been shown that the endogenous miRNAs can form complexes with proteins or be packaged in various lipid vesicles protecting them from abundant RNase in plasma [4], [19], [20], [21], [22]. To explore the stability of exogenous miRNAs and RNAs in circulation, we treated the plasma samples with DNAse, protease, Trixon X-100, and additional RNase before RNA isolation. Like endogenous miRNA (miR-16), the levels of specific exogenous miRNA (miR-263a-5p) and RNA (16S rRNA from *Pseudomonas putida*) were reduced significantly after Triton X-100, protease, RNase, and protease followed by RNase treatments (Figure S4). Adding additional RNase caused less reduction compared to protease followed by RNase treatments. This suggests that some of the exogenous RNA molecules, like endogenous miRNAs, are probably associated with protein and/or lipid complexes in circulation and a fraction of those complexes may not be tightly bound, such that the freeze thawing process or incubation at 37°C during enzyme treatment may release some of the protected RNAs.

### Exogenous RNA Sequences Observed in Plasma can Affect Cellular Gene Expression Pattern

While the functions of extracellular miRNAs are not fully understood, it has been demonstrated that certain cells can take up the miRNA contained in lipid vesicles, which results in changing the cell’s gene expression profile [19]. To explore the potential functions of exogenous RNAs in circulation, we transfected several synthetic, double-stranded mature microRNA-like molecules selected from observed exogenous miRNA sequences and some highly abundant exogenous sequences (bacterial rRNAs) that have potential to form pre-miRNA-like secondary structures (Figure S5) into a mouse, Dicer-deficient, fibroblast cell line. Because of the lack of the Dicer protein, a key RNAse III, miRNA processing enzyme, the Dicer deficient cells contain very much less mature miRNA compared to normal cells. This provides a good tool for studying the function of miRNAs. By introducing individual miRNA into these cells and avoiding multiple interactions of microRNA and mRNA (Wang et al in preparation) it is possible to interrogate the cells for mRNA levels, which are informative as to specific miRNA function. Based on microarray profiling results, it is clear that the expression profiles of a number of genes in the cells were affected by some of the exogenous RNA sequences. We verified the changes in levels of some of these affected genes’ mRNA by QPCR (Table S2 and Figure S6). The pathways enriched among those down-regulated genes are listed in Table 2. Two of the insect miRNAs, miR-263a-5p and bantam, did not produce any significant effects on the cellular transcriptome, which suggests that the process of transfection itself was not the cause of the observed gene expression changes. This observation suggests that RNA sequences in plasma might have some biological effects on human cells.

**Table 2:** Affected pathways by transfecting the synthetic exogenous RNA sequences.

Exogenous RNA Sequence	Species	Affected pathways	*P*-Value
**AE1:**GAACUGAAGAGUUUGAUCAUGG	16S rRNA from *Pseudomonas*	Apoptosis	3.90E-03
		Oocyte meiosis	1.50E-02
		Small cell lung cancer	1.60E-02
		RNA degradation	2.00E-02
		Proteasome	4.80E-02
		Pathways in cancer	5.40E-02
		Spliceosome	6.50E-02
		Pentose phosphate pathway	7.50E-02
		Huntington’s disease	9.90E-02
**AE2:**AUUUACUGUCUGAGCUGGGUGG	23S rRNA from *Rhodococcus*	Renal cell carcinoma	6.70E-03
		Chronic myeloid leukemia	8.40E-03
		Regulation of actin cytoskeleton	3.10E-02
		Neurotrophin signaling pathway	3.50E-02
		Tight junction	3.80E-02
		MAPK signaling pathway	5.80E-02
		Chemokine signaling pathway	8.00E-02
		ErbB signaling pathway	8.40E-02
		Focal adhesion	9.70E-02
**AE3:**CAGGCGUAGCCGAUGGACAACG	23S rRNA from *Rhodococcus*	Pathways in cancer	1.20E-02
		Adipocytokine signaling pathway	1.50E-02
		Pancreatic cancer	1.80E-02
		Focal adhesion	1.90E-02
		Endocytosis	7.20E-02
		Cell cycle	7.80E-02
		RNA degradation	8.00E-02
**AE4:**CGAAUAGGGCGAUCGUAGUGGC	23S rRNA from *Rhodococcus*	No enriched pathway	
**miR-263:**AAUGGCACUGGAAGAAUUCACGG	miR-263a from mosquito	No enriched pathway	
**Bantam:**UGAGAUCAUUGUGAAAGCUGAUU	Bantam from house fly	No enriched pathway	

### Exogenous RNA Sequences can be Detected in RISC Complex

It has been reported that cells in culture can take up microvesicles and internalize its molecular contents including RNA [23], [24]. Since some of the exogenous RNAs in circulation were packaged in lipid vesicles, we investigated whether the cellular machinery involved in small RNA function could incorporate exogenous RNA sequences. We compared the exogenous RNA spectrum between fetal bovine serum used in cell culture and intracellular RNA associated with RISC complex, immunoprecipitated by argonaute 2 (Ago2), a key component of the complex. Mass spectrometry based proteomic analysis on immunoprecipitated proteins revealed the presence of Ago2 along with several DDXs (DEAD box, helicase domain containing proteins), hnRNPs (heterogeneous nuclear ribonucleoprotiens) and RRMs (RNA recognition motif containing proteins) proteins as reported earlier [25] (Table S9). The presence of Ago2 in the immunoprecipitated protein mixture was also verified by Western blot (Figure S7).

**Figure 5:**
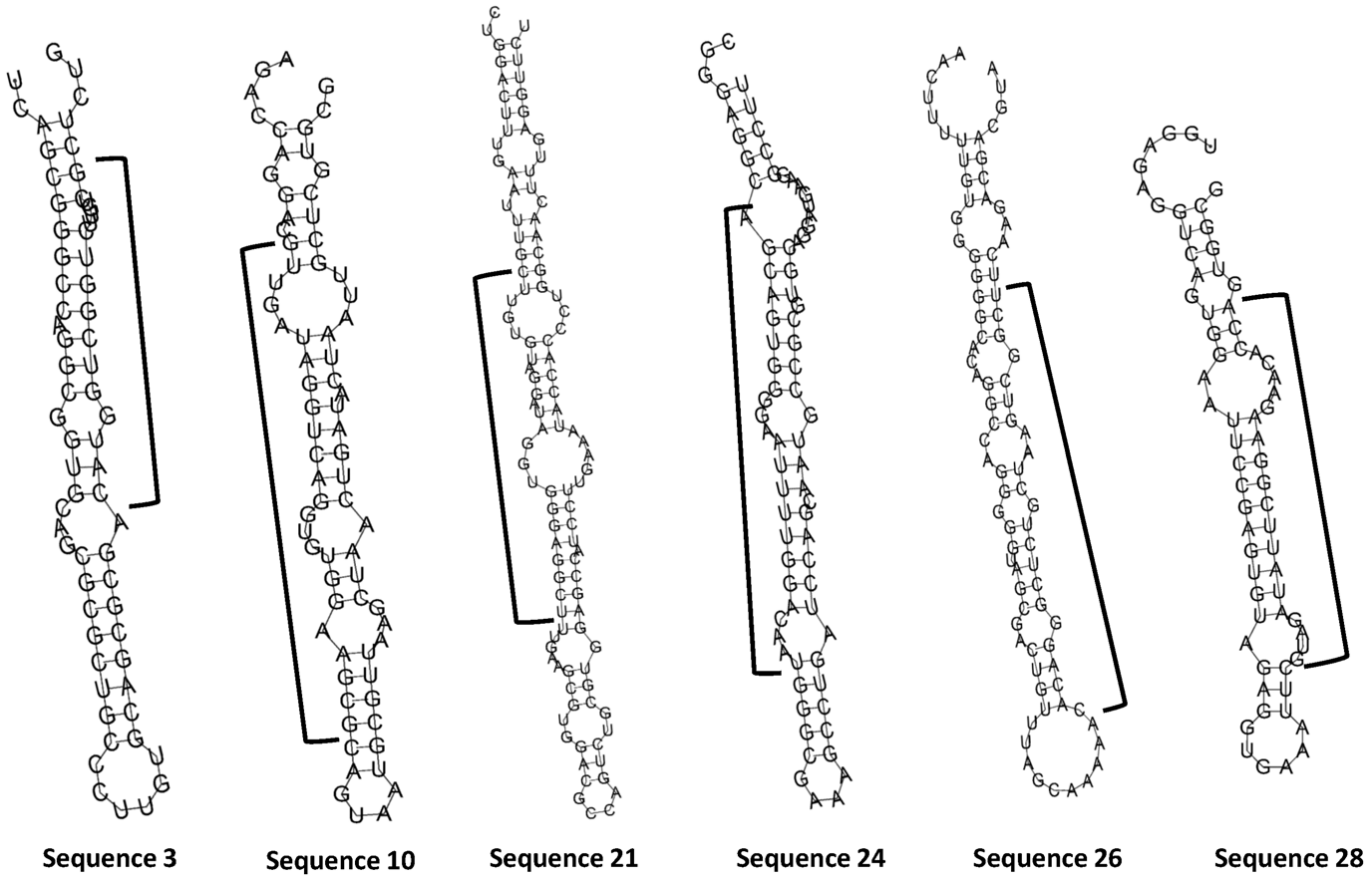
Examples of exogenous RNAs observed in fetal bovine serum and associated with RISC complex isolated from 293T cells that may generate miRNA precursor like hairpin structure. The regions indicated by black brackets lines were observed in sequencing results and listed on Table 4. RNAfold web server (www.rna.tbi.univie.ac.at/cgi-bin/RNAfold.cgi) was used to generate the putative secondary structure.

Compared to the total exogenous RNA in human plasma samples (Table S5), the exogenous RNA populations associated with RISC complex are significantly smaller (ranging from 4% to 16%, depending on search criteria) (Table 3). The exogenous RNA population in fetal bovine serum was even lower than found associated with the RISC complex. Looking carefully at the sequences we obtained, there are a number of sequences that mapped to various bacterial transcripts that were present in both the RISC complex and fetal bovine serum (examples see Table 4, full list on Table S10). Some of the regions contain these sequences can form miRNA precursor like hairpin structure (Figure 5). This observation further supports the suggestion that exogenous RNA sequences may influence the function of cells through a miRNA-like mechanism.

**Table 3:** Comparing sequence distribution between RNA associated with RISC complexes from 293T cells and fetal bovine serum using different mapping criteria.

Sample	293T cells Ago2 IP			Bovine Serum		
Search method	Strategy 0	Strategy 1	Strategy 2	Strategy 0	Strategy 1	Strategy 2
Endogenous Sequence	26.12%	37.43%	47.69%	8.67%	11.72%	13.50%
Bacteria Sequence^a^	13.00%	9.07%	3.79%	0.28%	0.07%	0.04%
Fungi Sequence^a^	0.26%	0.13%	0.06%	0.09%	0.04%	0.02%
Other Sequence^a^	2.45%	0.60%	0.31%	0.99%	0.21%	0.11%
Unmapped Sequences	58.16%	52.78%	48.14%	89.97%	87.96%	86.33%

a To increase the sequence mapping accuracy, we did not allow any sequence mismatch except in the endogenous sequence search step.

**Table 4:** Examples of identical exogenous sequences observed in fetal bovine serum used in culture and RISC complex from cultured cells.

Sequence ID	Sequence	Number of Reads Observed		Sequence Mapped to
		293T Ago2 IP	Fetal Bovine Serum	
Sequence 3	CATGGTCGGTCTTGTCGCTCTGG	2	2	*Acidovorax ebreus* Hypothetical protein
Sequence 10	GTTGATAGGTCAGGTGTGGAAGCG	12	8	*Variovoras paradoxus* EPS 23S rRNA
Sequence 21	CTTGTGTAGGATAGGTGGGAGGCT	2	1	*Pseudomonas fluorescens* 23S rRNA
Sequence 24	AGCAGTGGGGAATTTTGGACAAT	2	1	*Delftia sp*. Cs1-4 16S rRNA
Sequence 26	CACAGGGCTCTGCTAAGTCGGCT	2	1	*Sphingobium sp*.5S rRNA
Sequence 28	GTAGATATTCGGAAGAACACCA	1	1	Uncultured bacterium 16S rRNA

## Discussion

Since most of the circulating endogenous RNAs we identified originate from specific microbiome species, different conditions, benign and pathological, may skew the circulating RNA population. It may be surprising that we did not observe any significant differences in the general spectrum of endogenous and exogenous RNAs in circulation between samples from patients and normal individuals used in this study. This is probably caused by the high degree of heterogeneity of circulating RNA among individuals and the small sample size used. Imprecise diagnosis can also complicate the linkage between disease and circulating RNA population. Larger sample sizes with well documented pathological conditions or using animal models in a controlled environment will be used to shed further light on this observation.

It has long been thought that the human body is highly insulated from its environment by two major protective mechanisms, an active and dynamic immune system, and the body’s physical barrier represented by skin, gut epithelium, mucus membranes etc… While some sequences mapped to known human transcripts and other genomic sequences, we were surprised, even after very stringent screening criteria, to see that a significant proportion of the sequencing reads from human plasma samples in our study clearly originated from various microbes, insects and food sources (Figure 2a). We used simulated (Table S4) and public domain datasets (Figure 2c), and also examined several different types of samples, to ensure our sequence mapping protocol reliably assigns the endogenous sequences, and we employed a number of controls to eliminate the possibility of contamination in our samples and reagents. Treating the plasma samples with DNAse prior to RNA isolation has little or no effect on the levels of exogenous sequences, but the levels of these sequences decreased by about 2 fold when the plasma samples were treated with RNase (Figure S4). This provides support for the conclusion that most of the exogenous sequences in our results derive from RNA rather than DNA. To escape enzymatic degradation, these RNA molecules probably form complexes with proteins and/or lipid molecules, as has been previously reported for endogenous miRNAs, since treating the plasma samples with protease and Triton further decreased the levels of these exogenous RNA molecules (Figure S4).

The ability to accurately assign the origin of exogenous sequences is dependent on the availability of genome sequences from each species. Even though the genomes from a significant number of species have been determined in recent years, the number is insignificant compared to the number of species in the biota, particularly bacteria, archea and fungi. This includes many species in the human microbiome. Although we could not determine the origin of those exogenous RNA sequences with complete confidence, the diverse and numerous reads that showed perfect matches to a wide range of known microbial sequences suggest the spectrum of RNA in the blood appears to reflect the population of the gut, including the microbiome composition. Interestingly, samples derived from the human gastrointestinal tract were recently found to be significantly enriched in small RNAs when compared to microbial community samples derived from other environmental settings [26]. This finding along with the results of the present work suggest that the human gastrointestinal microbiota may disproportionately synthesizing small RNA molecules which are then reflected in human blood. In addition, the finding of a significant amount of RNA mapping to common cereal grains including corn, rice and wheat (Figure 4) clearly indicates that part of the RNA spectrum in the circulation is also provided directly from food intake.

The existence of exogenous RNAs in circulation is intriguing for several reasons. These molecules could possibly be molecular waste in the process of degradation and elimination by the body, and they could represent potential nutrients destined for further degradation and absorption. However, we cannot easily explain how such a large molecules as these RNAs must be enter the blood stream through the gut epithelium. Most of these RNAs are likely to be complexed with proteins and lipids, allowing them to escape RNase degradation. In addition, we note that these RNA molecules could influence cellular mRNA expression if they are taken up by cells. We demonstrated this by transfecting synthetic RNA sequences from some of the more abundant exogenous sequences we found in plasma samples. We also found the same (identical) bacteria sequences from fetal bovine serum in the RISC complex of cultured cell lines (Table 4 and Table S10). Even though the concentration of RNA we use in transfection (10 nM) is much higher compared to plasma (<0.3 pM based on an average of 100 ng/ml of RNA in plasma with an average of 100 nucleotide in length and the selected RNA sequence represents less than 0.01% of the total RNA population), we cannot exclude the possibility that certain cells in the body have an active uptake system which can pick up the circulating RNA (both endogenous and exogenous RNAs) at low concentrations. The recent finding of the rice miR-168a which has an estimated serum concentration in fM level in various mouse tissues further supports the possibility of an active uptake system for some cells to take up low concentration, circulating RNA [15] into their intracellular compartments. Although extracellular nucleic acids in plasma were discovered almost 60 years ago [27], the identification of stable extracellular miRNA in circulation supports the the fundamental idea of RNA-mediated signaling processes between cells. If some of the exogenous RNAs we find in circulation have the potential to influence cellular activity, individuals having different levels of these RNAs from normal food intake or from different microbiome populations could be affected by these differences in unexpected ways. The complex interactions of diet, microbiome and the cellular functions of the body, are affected in turn by genetics, which suggests new dimensions of the gene-environment interaction spectrum.

During the review process of this manuscript, Semenov et al reported the finding of exogenous RNAs that mapped to microbial sequences including *Escherichia, Acinetobacter, Propionibacterium* and others in normal human plasma using a SOLiD sequencing platform [28]. This independent study adds to the evidence we present here and supports the idea of exogenous RNA present in circulation as a common phenomenon in humans.

The finding of diverse exogenous RNA molecules in plasma, and their potential influence on cellular gene expression, raises several interesting questions about how humans interact with their environments and particularly with their gastrointestinal biota. Though the interaction between microbes and gut epithelium is yet to be fully understood, some sort of feedback signaling process might well be involved [6], [29]. Peptides and small chemicals have long been thought to be the two major types of signaling molecules between microbe and gut epithelium. The finding of microbial RNA in circulation now adds the possibility of an RNA-mediated human-microbiome interaction as an additional communication mechanism of this important axis for human health.

## Materials and Methods

### Ethics Statement

All protocols used in generating the mouse cell line were in compliance with federal guidelines and under the approval of Institute for Systems Biology’s Animal Care and Use Committee (01.01.10). Human samples used in this study were obtained according to the principles expressed in the Declaration of Helsinki, approved by the Ethics Review Committee at the Russian Oncological Research Center (PG-ONC 2003/1) and written informed consent was obtained from the patients.

### Sample and RNA Isolation

Plasma samples were obtained from Proteogenex (Culver City, CA). All samples were collected from donors with proper approvals from institutional review boards. The plasma was prepared from EDTA blood by centrifugation at 1,000×g for 15 minutes to separate the plasma and blood cells. Total RNA from plasma was extracted from 100 µl of the sample using the miRNeasy kit (Qiagen, Valencia, CA). The quality and quantity of the RNA were evaluated with Agilent 2100 Bioanalyzer (Santa Clara, CA) and NanoDrop 1000 spectrophotometer (Thermo Scientific, Wilmington, DE). In average, we obtained about 100 ng of RNA per ml of plasma. As a control we also obtained total RNA from Ambion (Life Technologies, Carlsbad, CA).

### NextGen Sequencing Library Preparation

NextGen sequencing libraries were prepared with small RNA sample preparation kits from Illumina (Illumina, San Diego, CA). The 3′ and 5′ adapters, and the reverse transcription primer were diluted in nuclease-free water to concentration specified by Illumina. RNA isolated from 200 µl of plasma (pooled from two 100 µl isolations) was concentrated and mixed with the diluted 3′ adapter in a final volume of 6 µl of nuclease free water. To eliminate secondary structures, the tube was incubated at 70°C for 2 minutes, then immediately cooled on ice. The ligation reaction was set by adding 1 µL of 10×T4 RNL2 reaction buffer, 0.8 µL of 100 mM MgCl_2_, 1.5 µL of T4 RNA ligase 2, and 0.5 µL of RNaseOUT RNase inhibitor (Life Technologies, Carlsbad, CA) and then incubated at 22°C for 1 hour. After ligating the 3′ adaptor, 1 µL of the 5′ adapter, 1 µL of 10 mM ATP, and 1 µL of T4 RNA Ligase were added, then incubated at 20°C for 4 hours.

For cDNA synthesis, 4 µL of RNA ligated with both 5′ and 3′ adaptors was mixed with 1 µL of diluted reverse transcription primer and incubated at 70°C for 2 minutes, then cooled on ice. 2 µL of 5× first-strand synthesis buffer, 0.5 µL of 12.5 mM dNTP mix, 1 µL of 100 mM DTT, and 0.5 µL of RNase OUT were added to the annealed primer-template mixture. The sample was then heated at 48°C for 3 minutes. One µL of SuperScript II Reverse Transcriptase was added to the sample and incubated at 44°C for 1 hour. The first-strand cDNA was then amplified with GX1 and GX2 primers using a condition as following: 98°C for 30 seconds, followed by 20 cycles of 10 seconds at 98°C, 30 seconds at 60°C, 15 seconds at 72°C, holding for 10 minutes at 72°C, then holding at 4°C. Since the amount of RNA in plasma is low, we did not use the small RNA-enriched fraction for sequencing library preparation; rather we selected and purified the cDNA through 6% Novex TBE PAGE gel (Life Technologies, Carlsbad, CA) a larger library insert size, covering 20 to 100 nucleotides in length. By doing so, we expected to get lower percentage of sequence reads for miRNA, but gain the ability to see the general spectrum of RNA in plasma including other ncRNAs and degraded messenger RNAs (mRNA). A 0.6 mL microcentrifuge tube was punctured 4–5 times using a 21-gauge needle and placed in a 2 mL microcentrifuge tube. The gel was stained with ethidium bromide then the gel fragments containing nucleotides of the desired length were excised and placed in the punctured 0.6 mL tube. The stacked tubes were centrifuged at 14,000 rpm for 2 minutes. 100 µL of gel elution buffer was added to the gel debris and incubated at room temperature overnight with agitation to elute the DNA. Following elution, the eluate and gel debris were transferred to the top of a Spin-X filter and centrifuged at 14000 rpm for 2 minutes to remove the gel debris. The DNA was purified by ethanol precipitation and resuspended in 10 µL of resuspension buffer. The quality and quantity of the library was assessed by using the Agilent 2100 Bioanalyzer with the DNA 1000 chip. The prepared library was then run on Illumina GenomeAnalyzer IIx at the genomic facility at the Institute for Systems Biology.

### NextGen Data Analysis

We obtained over 20 million sequence reads per sample with 35 cycle runs on Illumina Genome Analyzer IIx (Table S1). After trimming the adaptor sequences, removing low quality sequences, adaptor only sequences, and sequences containing only polyA, we generally had 2 to 4 million processed reads with an average length of 23 nucleotides.

A NextGen sequence read simulator, ART (http://bioinformatics.joyhz.com/ART/), was used to generate artificial transcriptome data from human, mouse, bovine and yeast. Transcript sequences from ENSEMBL and miRNA sequences from miRBase were combined and used as reference sequences. Illumina read error profile was selected for the program to generate artificial reads with either 23 or 35 nucleotides in length, from the reference sequences.

#### Sequence mapping scheme

The processed sequences were first screened against endogenous (human) sequence databases including known human miRNA, human transcripts, followed by human genomic sequence. To get complementary and efficient mapping results, the alignment tool Blast was used to search miRNA, and Bowtie was used to search other large databases. For the endogenous sequence mapping, except miRNA, we applied three different levels of error tolerance: 0 mismatch (termed Strategy 0), 1 mismatch (termed Strategy 1) and 2 mismatch (termed Strategy 2). The remaining unmapped sequences were then compared to sequences from the known human microbiome, miRNA sequences from other species, and the non-redundant nucleic acid sequence collection from NCBI. Due to the high sequence similarity for miRNA, we did not allow any sequence mismatch for both endogenous and exogenous miRNA mappings. We also did not allow any sequence mismatches for exogenous sequence mapping to increase the accuracy of mapping results (Figure 1). Species classification was based on NCBI Taxonomy database (http://www.ncbi.nlm.nih.gov/taxonomy).

### Transfection

The mouse Dicer deficient (DCR −/−) fibroblast cell line was generated from a conditional cre-lox Dicer allele, transgenic mouse available from JAX (http://jaxmice.jax.org/strain/006001.html) kindly provided by Dr. Jacques Peschon. Part of the RNase III domain encoded in the exon 23 of dicer gene was deleted following cre excision. DCR −/− cells were maintained in Dubecco’s modified Eagle’s medium with high glucose. The media was supplemented with 10% FBS, 1% non-essential amino acid, 1% Glutamax. The cells were routinely incubated at 37°C in a humidified atmosphere with 5% CO_2._

LipofectamineRNAiMAX was purchased from Invitrogen (Life Technologies, Carlsbad, CA). Exogenous RNA for transfection was obtained from Ambion (Life Technologies, Carlsbad, CA) and followed their custom miRNA design protocol. DCR−/− cells were seeded at a density of 1×10^5^ cells in 6-well tissue culture plates 24 h prior to transfect with 10 nM of synthetic RNAs. Cells exposed to transfection reagents only were used as control. After 24 hours in the transfection media, the cells were harvested for RNA isolation and the transfection efficiency was validated with QPCR.

### Microarray Analysis

The effects of exogenous RNAs on transcriptome were assessed by using the Agilent mouse 4 × 44K microarray (Agilent, Santa Clara, CA). Total RNAs were isolated with an miRNeasy column (Qiagen, Valencia, CA), and both Cy3 and Cy5-labeled cRNA samples were prepared with two color labeling kit (Agilent Technologies, Santa Clara, CA) and then hybridized at 65°C for 17 h. Signal intensity was calculated from digitized images captured by a scanner from Agilent (Santa Clara, CA), and data analysis was performed by using GeneSpring GX software (Agilent Technologies, Santa Clara, CA).

### Immunoprecipitation

HEK 293T cells were maintained in DMEM containing 10% FBS, 2 mM L-glutamine, 100 U/mL penicillin, and 100 µg/mL streptomycin (Life Technologies, Carlsbad, CA). For immunoprecipitation, cells were rinsed twice in ice-cold PBS and lysed for 30 min on ice in NP40 lysis buffer (Life Technologies, Carlsbad, CA) containing protease inhibitor cocktail (Thermo Fisher Scientific, Rockford, IL) and RNAse inhibitor (Promega, Madison, WI). Lysates were cleared by centrifugation at 13,000 rpm at 4°C for 20 min. Cleared lysates were transferred to new tubes and pre-adsorbed by incubating with Dynabeads protein G beads (Life Technologies, Carlsbad, CA) pre-blocked with RNAse-free BSA (Life Technologies, Carlsbad, CA) and yeast tRNA (Life Technologies, Carlsbad, CA) for 30 min at 4°C. Pre-adsorbed lysates were transferred to new tubes containing pre-blocked Dynabeads protein G beads coated with rabbit anti-Ago2 (clone C34C6; Cell Signaling Technology, Danvers, MA) antibodies and incubated overnight at 4°C with rocking. Beads were collected and washed several times with lysis buffer. RNA was isolated from washed beads using the miRNeasy kit (Qiagen, Valencia, CA) and prepared for sequencing.

The immunoprecipitated protein mixture was enzymatically digested with trypsin and desalted with C18 Ultramicrospin columns (The Nest Group, Southborough, MA). After drying in a speedvac (Thermo Fisher Scientific, Rockford, IL) the sample was re-suspended and analyzed on a high-performance linear ion trap LTQ-Orbitrap Hybrid mass spectrometer (Thermo Fisher Scientific, Waltham, MA). The MS/MS scans were searched against a human IPI database (version 3.38, decoy-combined) using X!Tandem search algorithm to determine peptide identity. A Proteinprophet probability score ≥0.7 (equals to ∼2.0% false positive rate) was set to filter the search data.

### Quantitative Polymerase Chain Reaction

The levels of the exogenous miRNA species were quantified by using a TaqMan microRNA assays (Life Technologies, Carlsbad, CA). cDNA was synthesized with a TaqMan microRNA reverse transcription kit (Life Technologies, Carlsbad, CA) from 2 µLof total RNA isolated from samples by a miRNeasy kit (Qiagen, Valencia, CA). Real-time RT-PCR was performed with StepOnePlus Real-Time PCR Systems (Life Technologies, Carlsbad, CA). The PCR conditions were as follows: initial enzyme activation and RNA denaturation at 95°C for 10 minutes, followed by 40 cycles of 95°C for 15s, 60°C for 1 min.

For other transcripts, SYBR based QPCR was used. For each sample, 6 µL of RNA was reverse transcribed using the miScript kit (Qiagen, Valencia, CA). For direct QPCR from plasma, 1 µLof plasma was mixed with 5 µL of RNase free water then heated at 95°C for 5 minutes prior to cDNA synthesis. cDNAs were diluted 2-fold and used 2 µLin SYBR-based QPCR reactions. The primers used in the study are listed in Table S2. The PCR fragments were cloned and sequenced to verify their origin.

To explore the stability of exogenous RNA in plasma, we treated the plasma with RNase A from Fementas (Fementas, Thermo Scientific, Wilmington, DE) at a concentration at 1 µg/ml, DNase I (Fermentas, Thermo Scientific, Wilmington, DE) at a concentration of 1 unit/ml, protease K (Fermentas, Thermo Scientific, Wilmington, DE) at a concentration of 0.05 mg/ml, 0.1% triton×100, or protease K for 20 minutes followed by additional RNase A at 1 ug/ml after heat inactivation of protease K at 70°C for 10 minutes prior for RNA isolation.

## Supporting Information

Figure S1 **miR-134 and let-7a (labeled on top) showing concentration differences between normal and patient plasma samples determined by sequencing (A) and QPCR (B).** The Y-axis represents the level of miRNA in either log 2 transformed read number (A) or 40-Ct value (B). The sample types and number of samples (number in parentheses) are indicated on the X-axis. The significance values (p value) from a t test are indicated.
(TIF)

Figure S2 **The level of exogenous RNA in plasma is not generated from contaminated plasma.** Primers specific for three different highly abundant ribosomal RNA species from *Ceratocystiopsis minuta* (fungus), *Pseudomonas putida* (bacterium) and human were used in RNA isolated from plasma directly (solid bars) and plasma that had been filtered by 0.2 uM filter (open bars).
(TIF)

Figure S3 **Some sequence reads from plasma are mapped to common yeast.** The number of processed reads (solid bars) and reads after removing tRNA and rRNA sequences (open bars) that mapped to common yeast is shown in the figure. The Y-axis represents the level of RNA in log 2 transformed read number and the identity of all 9 individuals is indicated on the X-axis.
(TIF)

Figure S4 **The relative changes of RNA concentrations after treating the plasma with DNase, RNase, Protease and triton X-100.** The plasma samples were treated with various conditions (indicated on the top of the figure) prior to RNA isolation. The Y-axis represents the relative concentration change compare to no treatment determined by QPCR. The data represent the average changes from 9 plasma samples. The black bars represent the changes of an endogenous miRNA, miR-16, the open bars are exogenous miRNA, miR-263 from mosquitos and the gray bars are the 16S rRNA from *Pseudomonas putida*.
(TIF)

Figure S5 **Exogenous RNAs that may generate miRNA precursor like hairpin structure were selected for transfection experiment.** The regions indicated by red lines were selected and used to generate synthetic RNA for transfection. RNAfold web server (www.rna.tbi.univie.ac.at/cgi-bin/RNAfold.cgi) was used to generate the secondary structure.
(TIF)

Figure S6 **The levels of several endogenous mRNAs can be affected by transfecting synthetic exogenous RNAs.** The identities of exogenous RNAs (AE1, AE2 and AE3) and endogenous genes are indicated on the top of the figure. The black bars are the changes of expression levels measured by microarray while gray bars represent the relative changes measured by QPCR (average of 3 independent measurements).
(TIF)

Figure S7 **The immunoprecipitation of Argonaute 2 protein (a) and validation by western blotting (b).** The size of molecular marker is labeled on the left.
(TIF)

Table S1 **Sample information.**
(DOCX)

Table S2 **Primer sequences used in the study.**
(DOCX)

Table S3 **Sequence distribution under different search criteria.**
(DOCX)

Table S4 **Sequence distribution using simulated RNA_seq data from different species.**
(DOCX)

Table S5 **Sequence distribution under different search criteria for human plasma samples.**
(DOCX)

Table S6 **Sequence distribution under different search criteria for different types of samples.**
(DOCX)

Table S7 **Sequence distribution under different search criteria for two public domain sequences.**
(DOCX)

Table S8 **Distribution of exogenous sequences mapped to microbiome based on kingdom and phylum.**
(DOCX)

Table S9 **List of proteins associated with argonaute 2 (Ago2).**
(DOCX)

Table S10 **List of identical exogenous sequences observed in fetal bovine serum used in culture and RISC complex from cultured cells.**
(DOCX)
