# Supplementary figures and images for: The Complex Exogenous RNA Spectra in Human Plasma: An Interface with Human Gut Biota?
Source: PLoS One. 2012 Dec 10;7(12):e51009. doi: 10.1371/journal.pone.0051009 (PMC3519536; doi:10.1371/journal.pone.0051009)

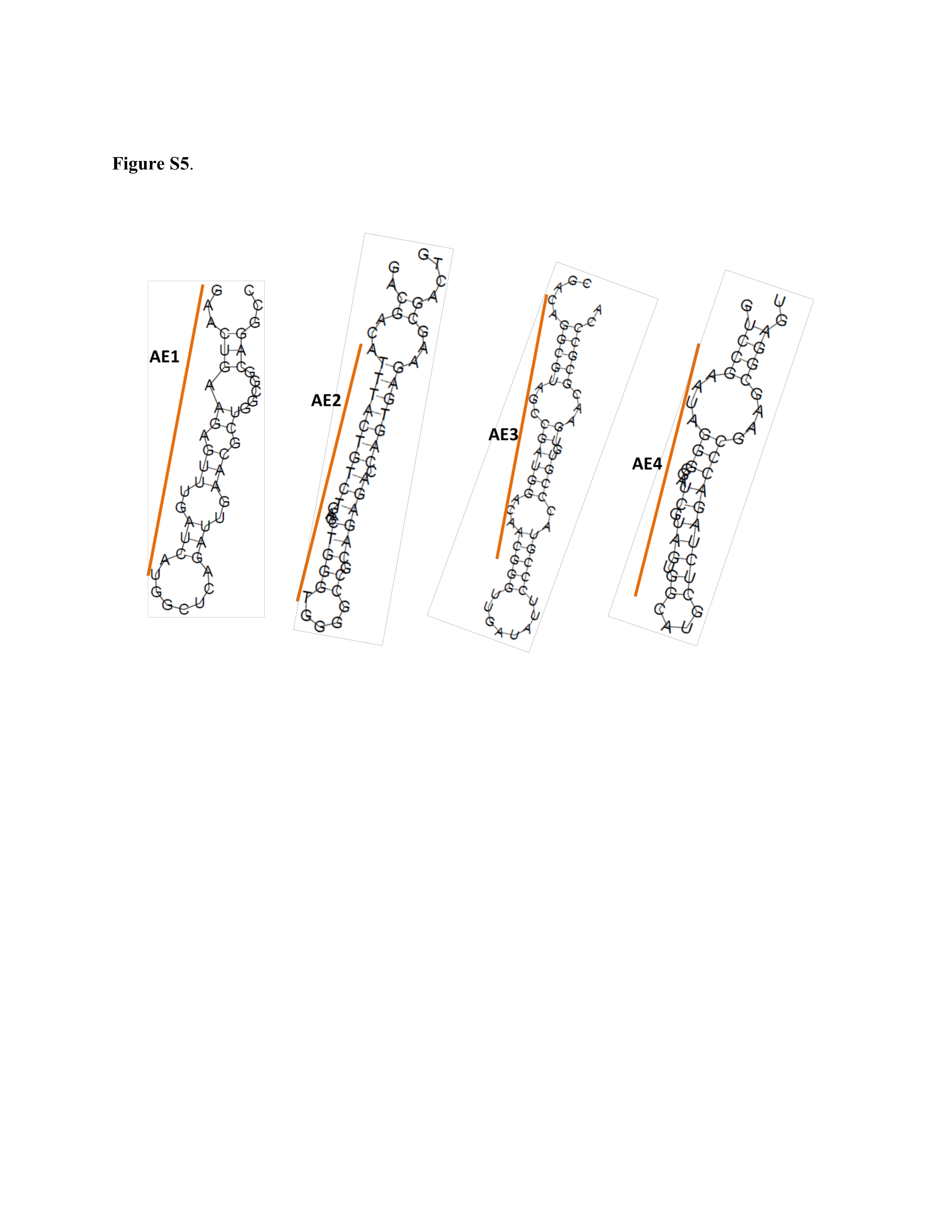

Supplement: Figure S5 — Exogenous RNAs that may generate miRNA precursor like hairpin structure were selected for transfection experiment. The regions indicated by red lines were selected and used to generate synthetic RNA for transfection. RNAfold web server (www.rna.tbi.univie.ac.at/cgi-bin/RNAfold.cgi) was used to generate the secondary structure. (TIF) [file pone.0051009.s005.tif]
