# Supplementary material for: The Complex Exogenous RNA Spectra in Human Plasma: An Interface with Human Gut Biota?
Source: PLoS One. 2012 Dec 10;7(12):e51009. doi: 10.1371/journal.pone.0051009 (PMC3519536; doi:10.1371/journal.pone.0051009)
Supplement: Table S1 — Sample information. (DOCX) [file pone.0051009.s008.docx]

**Table S1**.

| **Sample ID** | **Gender** | **Age** | **Ethnic background** | **Classification** | **Disease stage** | **Number of reads** | **Processed reads** |
| --- | --- | --- | --- | --- | --- | --- | --- |
| D3340P | M | 58 | Caucasian | Normal |  | 27,002,901 | 5,085,400 |
| D3176P | F | 52 | Caucasian | Normal |  | 27,957,185 | 4,933,712 |
| D3142P | F | 54 | Caucasian | Normal |  | 28,214,261 | 4,826,682 |
| 022273P | F | 52 | Caucasian | Colorectal cancer | I | 21,132,674 | 2,183,460 |
| 022163P | F | 56 | Caucasian | Colorectal cancer | III | 23,547,368 | 2,879,950 |
| 022299P | M | 46 | Caucasian | Colorectal cancer | III | 22,729,858 | 1,351,471 |
| 93163P | M | 67 | Caucasian | Ulcerative colitis | 10 ^(a)^ | 20,626,993 | 2,638,989 |
| 93164P | M | 58 | Caucasian | Ulcerative colitis | 11 ^(a)^ | 18,186,259 | 2,807,184 |
| 93166P | F | 48 | Caucasian | Ulcerative colitis | 11 ^(a)^ | 28,426,819 | 3,378,222 |
| ***(a):*** *Based on Mayo Scoring System for Assessment of Ulcerative Colitis Activity* | | | |  |  |  |  |
