# Supplementary material for: The Complex Exogenous RNA Spectra in Human Plasma: An Interface with Human Gut Biota?
Source: PLoS One. 2012 Dec 10;7(12):e51009. doi: 10.1371/journal.pone.0051009 (PMC3519536; doi:10.1371/journal.pone.0051009)
Supplement: Table S2 — Primer sequences used in the study. (DOCX) [file pone.0051009.s009.docx]

**Table S2**.

| **Gene and Species** | **Forward Primer** | **Reverse Primer** | |
| --- | --- | --- | --- |
| Human 28S rRNA | GTCGGGGTTTCGTACGTAGCAGAGC | GTCACGCACCGCACGTTCGTGG |  |
| Pseudomonas putida 16S RNA | AATACTAATTGCCCGTGAGGCTTGA | AAGAGATAACCGCGAAAGCATCTAAG |  |
| Ceratocystiopsis minuta 18S RNA | GCCCGTCGCTACTACCGATTGAATG | TCAACTTGCGTTGATTACGTCC |  |
| Human cytochrome c oxidase subunit VIIIA (Cox8 | CGTGGTCGTTCCGCGCCGTCA | CCGCGCCGGCTTCGAGTGGA |  |
| Human mitochondrial ribosomal protein S25 (Mrps25) | CGCTGGTCCGCGGCTTGAGGAG | TGCAGCGTACGGCGGATCGGGA |  |
| Human caveolin 2 (Cav2) | GCCACGATGGGGCTGGAGACCG | CGCCACTGTGGTGGCTGTAGGCGT |  |
| Human strawberry notch homolog 1 (Sbno1) | GCTGGCCTTGCCACCCCTACGC | GTGCGGGAGTGGACATCGGCTCTGG |  |
| Human leucine rich repeat containing 8 family, member A (Lrrc8a) | CCCACCCCCTGGCCACGCTCTT | AGGGAGCGCCGCAGCATCCAC |  |
| Human chloride intracellular channel 1 (Clic1) | GCGCCGAAGGGACAGCCCTGAGT | ACTGGGAGGGGCCGGGGCAA |  |
