# Supplementary material for: The Complex Exogenous RNA Spectra in Human Plasma: An Interface with Human Gut Biota?
Source: PLoS One. 2012 Dec 10;7(12):e51009. doi: 10.1371/journal.pone.0051009 (PMC3519536; doi:10.1371/journal.pone.0051009)
Supplement: Table S3 — Sequence distribution under different search criteria. (DOCX) [file pone.0051009.s010.docx]

**Table S3.**

| **Category** | **0 mismatch** | | |  | **1 mismatch** | | |  | **2 mismatch** | | |
| --- | --- | --- | --- | --- | --- | --- | --- | --- | --- | --- | --- |
|  | Normal (3) **^b^** | Colorectal Cancer (3) **^b^** | Ulcerative colitis (3) **^b^** |  | Normal (3) **^b^** | Colorectal Cancer (3) **^b^** | Ulcerative colitis (3) **^b^** |  | Normal (3) **^b^** | Colorectal Cancer (3) **^b^** | Ulcerative colitis (3) **^b^** |
| Human microRNA **^a^** | 1.43% | 1.25% | 1.63% |  | 1.43% | 1.25% | 1.63% |  | 1.43% | 1.25% | 1.63% |
| Human transcript | 2.78% | 2.51% | 2.55% |  | 19.27% | 18.09% | 19.32% |  | 42.30% | 40.33% | 43.43% |
| Human genome | 8.41% | 7.86% | 8.41% |  | 19.24% | 18.41% | 19.55% |  | 14.98% | 14.87% | 15.10% |
| Unmapped sequence | 87.38% | 88.38% | 87.42% |  | 60.06% | 62.25% | 59.51% |  | 41.29% | 43.55% | 39.85% |

1. Due to high sequence similarity for various miRNA species, we did not allow any sequence mismatch in miRNA alignment.
2. Numbers in parentheses represents number of samples in each group.
