# Supplementary material for: The Complex Exogenous RNA Spectra in Human Plasma: An Interface with Human Gut Biota?
Source: PLoS One. 2012 Dec 10;7(12):e51009. doi: 10.1371/journal.pone.0051009 (PMC3519536; doi:10.1371/journal.pone.0051009)
Supplement: Table S4 — Sequence distribution using simulated RNA_seq data from different species. (DOCX) [file pone.0051009.s011.docx]

**Table S4**.

| **Sample** | | **Human** | | |  | **Mouse** | | |  | **Bovine** | | |  | **Yeast** | | |
| --- | --- | --- | --- | --- | --- | --- | --- | --- | --- | --- | --- | --- | --- | --- | --- | --- |
| Category | Sequence length (nt) | 0 mismatch | 1 mismatch | 2 mismatch |  | 0 mismatch | 1 mismatch | 2 mismatch |  | 0 mismatch | 1 mismatch | 2 mismatch |  | 0 mismatch | 1 mismatch | 2 mismatch |
| Endogenous miRNA | 23 | 0.05% | 0.05% | 0.05% |  | 0.07% | 0.07% | 0.07% |  | 0.04% | 0.04% | 0.04% |  |  |  |  |
| Endogenous transcript | 23 | 87.93% | 99.23% | 99.89% |  | 87.88% | 99.21% | 99.89% |  | 87.84% | 99.18% | 99.85% |  | 87.82% | 99.27% | 99.98% |
| Endogenous genome | 23 | 0.12% | 0.02% | 0.01% |  | 0.08% | 0.02% | 0.01% |  | 0.07% | 0.02% | 0.01% |  | 0.00% | 0.00% | 0.00% |
| Unmapped Sequence | 23 | 0.31% | 0.00% | 0.00% |  | 0.17% | 0.00% | 0.00% |  | 0.31% | 0.02% | 0.01% |  | 0.09% | 0.00% | 0.00% |
| Endogenous miRNA | 35 | 0.02% | 0.02% | 0.02% |  | 0.01% | 0.01% | 0.01% |  | 0.03% | 0.03% | 0.03% |  |  |  |  |
| Endogenous transcript | 35 | 60.90% | 91.54% | 98.75% |  | 60.92% | 91.56% | 98.76% |  | 60.88% | 88.95% | 96.02% |  | 60.74% | 91.36% | 98.72% |
| Endogenous genome | 35 | 0.17% | 0.05% | 0.01% |  | 0.09% | 0.03% | 0.01% |  | 0.09% | 0.03% | 0.01% |  | 0.00% | 0.00% | 0.00% |
| Unmapped Sequence | 35 | 38.91% | 8.39% | 1.22% |  | 38.98% | 8.40% | 1.22% |  | 39.00% | 10.99% | 3.94% |  | 39.26% | 8.64% | 1.28% |
