# Supplementary material for: The Complex Exogenous RNA Spectra in Human Plasma: An Interface with Human Gut Biota?
Source: PLoS One. 2012 Dec 10;7(12):e51009. doi: 10.1371/journal.pone.0051009 (PMC3519536; doi:10.1371/journal.pone.0051009)
Supplement: Table S6 — Sequence distribution under different search criteria for different types of samples. (DOCX) [file pone.0051009.s013.docx]

**Table S6.**

| **Sample** | **Mouse Plasma (2) ^a^** | | | | | | | |  | | **Human Lung** | | | | | |  | | **Bovine Milk** | | | |
| --- | --- | --- | --- | --- | --- | --- | --- | --- | --- | --- | --- | --- | --- | --- | --- | --- | --- | --- | --- | --- | --- | --- |
| Search method | | Strategy 0 | Strategy 1 | | Strategy 2 | |  | | | Strategy 0 | | Strategy 1 | Strategy 2 | |  | Strategy 0 | | | | Strategy 1 | Strategy 2 |  |
| Endogenous Sequence | | 28.37% | | 53.02% | | 67.59% | |  | | 48.54% | | 53.05% | 54.62% |  | | | | 10.52% | | 34.78% | 57.51% |  |
| Bacteria Sequence ^b^ | | 11.34% | | 5.40% | | 3.10% | |  | | 0.15% | | 0.06% | 0.05% |  | | | | 60.43% | | 41.72% | 26.04% |  |
| Fungi Sequence ^b^ | | 7.05% | | 1.21% | | 0.63% | |  | | 0.56% | | 0.41% | 0.29% |  | | | | 0.67% | | 0.19% | 0.06% |  |
| Other Sequence ^b^ | | 9.85% | | 4.73% | | 3.15% | |  | | 1.37% | | 0.21% | 0.08% |  | | | | 3.09% | | 1.57% | 1.01% |  |
| Unmapped Sequence | | 43.39% | | 35.64% | | 25.53% | |  | | 49.38% | | 46.27% | 44.96% |  | | | | 25.29% | | 21.74% | 15.38% |  |

1. Numbers in parentheses represent number of samples in each group.
2. To increase the sequence mapping accuracy, we did not allow any sequence mismatch except in the endogenous sequence search step.
