# Supplementary material for: The Complex Exogenous RNA Spectra in Human Plasma: An Interface with Human Gut Biota?
Source: PLoS One. 2012 Dec 10;7(12):e51009. doi: 10.1371/journal.pone.0051009 (PMC3519536; doi:10.1371/journal.pone.0051009)
Supplement: Table S7 — Sequence distribution under different search criteria for two public domain sequences. (DOCX) [file pone.0051009.s014.docx]

**Table S7**.

| **Sample** | **Human Serum (SRR332232)^a^** | | | |  | **Yeast (SRR014350)^a^** | | | | | |
| --- | --- | --- | --- | --- | --- | --- | --- | --- | --- | --- | --- |
| Search Method | Strategy 0 | Strategy 1 | Strategy 2 | |  | Strategy 0 | | Strategy 1 | | | Strategy 2 |
| Endogenous Sequence | 15.33% | 48.75% | 70.78% |  | | | 16.01% | | 27.16% | 36.16% | |
| Bacteria Sequence ^b^ | 7.79% | 1.09% | 0.04% |  | | | 0.28% | | 0.02% | 0.00% | |
| Fungi Sequence ^b^ | 1.47% | 0.46% | 0.08% |  | | | 0.11% | | 0.11% | 0.11% | |
| Other Sequence ^b^ | 50.96% | 28.14% | 12.66% |  | | | 0.09% | | 0.03% | 0.03% | |
| Unmapped Sequence | 24.45% | 21.56% | 16.44% |  | | | 83.51% | | 72.68% | 63.70% | |

1. Numbers in parentheses are the access numbers.
2. To increase the sequence mapping accuracy, we did not allow any sequence mismatch except in the endogenous sequence search step.
