# Supplementary material for: The Complex Exogenous RNA Spectra in Human Plasma: An Interface with Human Gut Biota?
Source: PLoS One. 2012 Dec 10;7(12):e51009. doi: 10.1371/journal.pone.0051009 (PMC3519536; doi:10.1371/journal.pone.0051009)
Supplement: Table S8 — Distribution of exogenous sequences mapped to microbiome based on kingdom and phylum. (DOCX) [file pone.0051009.s015.docx]

**Table S8**.

|  | |  | **Mapped sequence (log 10)** | | | | | | |  | | | **Mapped sequence removed tRNA and rRNA (log 10)** | | | | |  |
| --- | --- | --- | --- | --- | --- | --- | --- | --- | --- | --- | --- | --- | --- | --- | --- | --- | --- | --- |
| **Kingdom** | **Phylum** | | **Normal** | | **Colorectal cancer** | | | **Ulcerative colitis** | |  | | **Normal** | | | **Colorectal cancer** | | **Ulcerative colitis** | |
| **Bacteria** | *Proteobacteria* | | 4.62 | | 4.69 | | | 4.59 | |  | | 4.22 | | | 4.29 | | 4.22 | |
|  | *Bacteroidetes* | | 4.14 | | 4.18 | | | 4.17 | |  | | 3.74 | | | 3.79 | | 3.77 | |
|  | *Firmicutes* | | 3.76 | | 3.84 | | | 3.77 | |  | | 3.66 | | | 3.75 | | 3.68 | |
|  | *Planctomycetes* | | 3.63 | | 3.63 | | 3.62 | |  | | 2.29 | | | 2.28 | | 2.26 | |  |
|  | *Actinobacteria* | | 3.42 | | 3.49 | | 3.44 | |  | | 3.27 | | | 3.34 | | 3.28 | |  |
|  | *Cyanobacteria* | | 3.18 | | 3.37 | | 3.23 | |  | | 1.81 | | | 2.07 | | 1.9 | |  |
|  | *Acidobacteria* | | 2.92 | | 2.94 | | 2.93 | |  | | 1.87 | | | 1.89 | | 1.84 | |  |
|  | *Verrucomicrobia* | | 2.91 | | 2.94 | | 2.92 | |  | | 1.76 | | | 1.81 | | 1.78 | |  |
|  | *Synergistetes* | | 2.67 | | 2.71 | | 2.69 | |  | | 2.64 | | | 2.68 | | 2.66 | |  |
|  | *Spirochaetes* | | 2.55 | | 2.62 | | 2.63 | |  | | 2.31 | | | 2.38 | | 2.39 | |  |
|  | *Fusobacteria* | | 2.51 | | 2.55 | | 2.52 | |  | | 2.48 | | | 2.51 | | 2.48 | |  |
|  | *Chloroflexi* | | 2.37 | | 2.45 | | 2.44 | |  | |  | | |  | |  | |  |
|  | *Deferribacteres* | | 2.23 | | 2.31 | | 2.3 | |  | |  | | |  | |  | |  |
|  | *Fibrobacteres* | | 2.18 | | 2.27 | | 2.33 | |  | |  | | |  | |  | |  |
|  | *Deinococcus-Thermus* | | 2.18 | | 2.27 | | 2.26 | |  | |  | | |  | |  | |  |
|  | *Elusimicrobia* | | 2.15 | | 2.21 | | 2.16 | |  | |  | | |  | |  | |  |
|  | *Nitrospirae* | | 2.16 | | 2.18 | | 2.17 | |  | |  | | |  | |  | |  |
|  | *Tenericutes* | | 2.11 | | 2.2 | | 2.15 | |  | |  | | |  | |  | |  |
|  | *Gemmatimonadetes* | | 2.01 | | 2.09 | | 2.1 | |  | | 1.69 | | | 1.79 | | 1.8 | |  |
|  | *Chlamydiae* | | 1.9 | | 1.95 | | 1.99 | |  | | 1.42 | | | 1.47 | | 1.53 | |  |
|  | *Aquificae* | | 1.88 | | 1.95 | | 1.95 | |  | |  | | |  | |  | |  |
|  | *Thermotogae* | | 1.87 | | 1.95 | | 1.95 | |  | | 1.16 | | | 1.21 | | 1.26 | |  |
|  | *Chlorobi* | | 1.86 | | 1.94 | | 1.94 | |  | |  | | |  | |  | |  |
|  | *Dictyoglomi* | | 1.87 | | 1.92 | | 1.91 | |  | |  | | |  | |  | |  |
|  | *Armatimonadetes* | | 1.87 | | 1.88 | | 1.9 | |  | |  | | |  | |  | |  |
|  | *Thermodesulfobacteria* | | 1.48 | | 1.6 | | 1.58 | |  | |  | | |  | |  | |  |
|  | *Chrysiogenetes* | | 1.08 | | 1.14 | | 1.07 | |  | |  | | |  | |  | |  |
|  | *Lentisphaerae* | | 1.03 | |  | | 1.07 | |  | |  | | |  | |  | |  |
| **Archaea** | *Euryarchaeota* | | 1.68 | | 1.79 | | 1.8 | |  | | 1.19 | | | 1.34 | | 1.3 | |  |
|  | *Crenarchaeota* | |  | | 1.33 | | 1.16 | |  | |  | | |  | |  | |  |
| **Fungi** | *Ascomycota* | | 5.16 | | 5.13 | | 5.13 | |  | | 4.72 | | | 4.7 | | 4.69 | |  |
|  | *Basidiomycota* | | 4.3 | | 4.27 | | 4.29 | |  | | 3.68 | | | 3.65 | | 3.67 | |  |
|  | *Glomeromycota* | | 3.95 | | 3.96 | | 3.95 | |  | | 2.51 | | | 2.5 | | 2.54 | |  |
|  | *Chytridiomycota* | | 3.58 | | 3.55 | | 3.51 | |  | | 2.92 | | | 2.79 | | 2.78 | |  |
|  | *Blastocladiomycota* | | 2.57 | | 2.72 | | 2.64 | |  | | 1.87 | | | 2.26 | | 1.92 | |  |
|  | *Neocallimastigomycota* | | | 2.32 | | 2.32 | | 2.52 | |  | | 1.08 | | | 1.18 | | 1.12 | |
|  | *Microsporidia* | | 1.57 | | 1.4 | | 1.41 | |  | |  | | |  | |  | |  |
