# Supplementary material for: The Complex Exogenous RNA Spectra in Human Plasma: An Interface with Human Gut Biota?
Source: PLoS One. 2012 Dec 10;7(12):e51009. doi: 10.1371/journal.pone.0051009 (PMC3519536; doi:10.1371/journal.pone.0051009)
Supplement: Table S9 — List of proteins associated with argonaute 2 (Ago2). (DOCX) [file pone.0051009.s016.docx]

**Table S9**.

| **Protein type** | **Protein name** |
| --- | --- |
| DEAD/DEAH box containing protiens | DDX1, DDX21, DDX3X, DDX41, DDX5 |
| Heterogeneous neclear ribonucleoprotein | HNRNPK, HNRNPL, HNRNPM |
| RNA binding protein | Rbm12b, YBX1, NCL, NPM1, EIF2S1, EIF2C2 (AGO2), PABPC4, PABPC1, SFPQ, NONO,TUT1 |
