# Supplementary material for: The Complex Exogenous RNA Spectra in Human Plasma: An Interface with Human Gut Biota?
Source: PLoS One. 2012 Dec 10;7(12):e51009. doi: 10.1371/journal.pone.0051009 (PMC3519536; doi:10.1371/journal.pone.0051009)
Supplement: Table S10 — List of identical exogenous sequences observed in fetal bovine serum used in culture and RISC complex from cultured cells. (DOCX) [file pone.0051009.s017.docx]

**Table S10**.

| **Sequence ID** | **Sequence** | **Number of reads observed** | | **Sequence Mapped to** |
| --- | --- | --- | --- | --- |
|  |  | **293T Ago2 IP** | **Fetal Bovine Serum** |  |
| Sequence 01 | TGAAAGTAGGTCATCGTCAGGCT | 45 | 1 | Achromobacter piechaudii ATCC 43553 genomic scaffold SCAFFOLD1 |
| Sequence 02 | CGAACTGTAGAGTTTGATCCTGGC | 35 | 23 | Alicycliphilus denitrificans BC chromosome |
| Sequence 03 | CATGGTCGGTCTTGTCGCTCTGG | 2 | 2 | Acidovorax ebreus Hypothetical protein |
| Sequence 04 | AAACTGAAGAGTTTGATCATGGC | 25 | 2 | Acinetobacter haemolyticus ATCC 19194 genomic scaffold SCAFFOLD7 |
| Sequence 05 | AACTGAAGAGTTTGATCATGGC | 6 | 4 | Acinetobacter haemolyticus ATCC 19194 genomic scaffold SCAFFOLD7 |
| Sequence 06 | AATCTGAGGGTCGGACGTTCGAATCGTTCCGGGCG | 10 | 1 | Bradyrhizobium sp. BTAi1 chromosome |
| Sequence 07 | GAACTATAGAGTTTGATCCTGGCTCAGATTGAACG | 10 | 2 | Comamonas testosteroni CNB-2 |
| Sequence 08 | GAACTGTAGAGTTTGATCCTGGCTCAGATTGAACG | 9 | 2 | Alicycliphilus denitrificans K601 chromosome |
| Sequence 09 | GAACTGTAGAGTTTGATCCTGGC | 8 | 7 | Acidovorax ebreus TPSY chromosome |
| Sequence 10 | GTTGATAGGTCAGGTGTGGAAGCG | 12 | 8 | Variovoras paradoxus EPS 23S rRNA |
| Sequence 11 | GCTAAGTGGGAAAGGATGTGGAG | 1 | 1 | Micrococcus luteus 23S rRNA |
| Sequence 12 | ATGTGTAGGATAGGTGGGAGACTTTGAAGCTGGGA | 7 | 2 | 23S ribosomal RNA\| [locus_tag=MHR_r002] |
| Sequence 13 | CGGATTCCCGTGTGAAAGTAGGTCATCGCCAGGCT | 7 | 1 | Ralstonia sp. 5_7_47FAA genomic scaffold supercont1.15 |
| Sequence 14 | GAACTGAAGAGTTTGATCCTGGCTCAGATTGAACG | 7 | 1 | Ralstonia sp. 5_7_47FAA genomic scaffold supercont1.15 |
| Sequence 15 | GTGTAGGATAGGTGGGAGACTTTGAAGCTGGGACG | 7 | 1 | 23S ribosomal RNA\| [locus_tag=MHR_r002] |
| Sequence 16 | AAACTGAAGAGTTTGATCATGGCTCAGATTGAACG | 6 | 4 | Acinetobacter haemolyticus ATCC 19194 genomic scaffold SCAFFOLD7 |
| Sequence 17 | CGCGGGATAGAGCAGCCTGGTAGCTCGTCGGGCTC | 6 | 1 | Met tRNA\| [locus_tag=Ava_R0004] |
| Sequence 18 | CGGGTTCCCGTGTGAAAGTAGGTCATCGTCAGGCT | 6 | 2 | 5S ribosomal RNA\| [locus_tag=Dtpsy_R0016] |
| Sequence 19 | GAGTTTGATCCTGGCTCAGAACGAACGCTGGCGGC | 6 | 3 | Treponema phagedenis F0421 genomic scaffold Scfld1052 |
| Sequence 20 | GGTTAGTCAGGGCCTAAGATGAGGCAGAAATGCAT | 6 | 2 | 23S ribosomal RNA\| [locus_tag=MHR_r002] |
| Sequence 21 | CTTGTGTAGGATAGGTGGGAGGCT | 2 | 1 | Pseudomonas fluorescens 23S rRNA |
| Sequence 22 | CTTGTCGGGCGTGTAGTAGGAG | 1 | 2 | Bradyrhizobium sp. Glutathione synthase |
| Sequence 23 | TGAACTGAAGAGTTTGATCCTGGCTCAGATTGAAC | 5 | 2 | Ralstonia sp. 5_7_47FAA genomic scaffold supercont1.15 |
| Sequence 24 | AGCAGTGGGGAATTTTGGACAAT | 2 | 1 | Delftia sp. Cs1-4 16S rRNA |
| Sequence 25 | CTGGACCTGTACTGACGCTCATGC | 2 | 1 | Variovoras paradoxus EPS 16S rRNA |
| Sequence 26 | CACAGGGCTCTGCTAAGTCGGCT | 2 | 1 | Sphingobium sp.5S rRNA |
| Sequence 27 | AGAGTAGGTCGTCGCCAGGCA | 2 | 1 | Sphingobium chlorophenolicum 23S rRNA |
| Sequence 28 | GTAGATATTCGGAAGAACACCA | 1 | 1 | Uncultured bacterium 16S rRNA |
| Sequence 29 | GGGATTGTAGTTCAATTGGTTA | 3 | 1 | Asp tRNA\| [locus_tag=MAE_t001] |
| Sequence 30 | TAAACTGAAGAGTTTGATCATGGC | 3 | 1 | Acinetobacter haemolyticus ATCC 19194 genomic scaffold SCAFFOLD7 |
| Sequence 31 | TAACTGAAGAGTTTGATCATGGCTCAGATTGAACG | 3 | 1 | Acinetobacter radioresistens SK82 contig00089 |
| Sequence 32 | TCTGAACATGGGGGGACCACCCTCCAAGCCTAAAT | 3 | 2 | Roseomonas cervicalis ATCC 49957 genomic scaffold SCAFFOLD3 |
| Sequence 33 | TTAACTGAAGAGTTTGATCATGGC | 3 | 3 | Acinetobacter radioresistens SK82 contig00089 |
| Sequence 34 | AGAGAACTCAGGAGAAGGAACTCGGCAAATTGACA | 2 | 2 | 23S ribosomal RNA\| [locus_tag=Pnap_R0029] |
| Sequence 35 | CAAGACCAGGACGTTGATAGGTCAGGTGTGGAAGC | 2 | 1 | 23S ribosomal RNA\| [locus_tag=Bphy_R0016] |
| Sequence 36 | CGGAATTACTGGGCGTAAAGC | 2 | 1 | Cardiobacterium hominis ATCC 15826 genomic scaffold SCAFFOLD2 |
| Sequence 37 | CTAATCGATCGAGGACTTAACC | 2 | 1 | Enterococcus faecium TX0133a01 E_faeciumTX0133a01-1.0_Cont36.1 |
| Sequence 38 | CTGGTCGGACGTCTGGATAGCCTTGGAATTCATCT | 2 | 1 | Acidovorax sp. JS42 chromosome |
| Sequence 39 | GCGGGTTCCCGTGTGAAAGTAGGTCATCGTCAGGC | 2 | 1 | 5S ribosomal RNA\| [locus_tag=Alide_R0062] |
| Sequence 40 | GTGCGGGTTCCCGTGTGAAAGTAGGTCATCGTCAG | 2 | 1 | 5S ribosomal RNA\| [locus_tag=Alide_R0062] |
| Sequence 41 | TAATCGGTCGAGGACTTAACC | 2 | 3 | Enterococcus faecalis TX0645 genomic scaffold Scfld48 |
| Sequence 42 | TAGCGGAACGCTCTGGAAAGTGC | 2 | 1 | 23S ribosomal RNA\| [locus_tag=PSEBR_23r4] |
| Sequence 43 | TCACTGTAGGGGGTAGAGCACTGTTTCGGCTAGGG | 2 | 1 | 23S ribosomal RNA\| [locus_tag=PSEBR_23r4] |
| Sequence 44 | TCCTCGATAGCTCAGTCGGTAGAGCGCCGGACTGT | 2 | 1 | Asn tRNA\| [locus_tag=Tmz1t_R0040] |
| Sequence 45 | TTAACTGGAGAGTTTGATCCTGGC | 2 | 1 | 16S ribosomal RNA\| [locus_tag=sce_rRNA_16_4] |
| Sequence 46 | TTCTTGACGACCATAGAGCATTGGAACCACCTGAT | 2 | 3 | ribosomal RNA 5s\| [locus_tag=PSEEN_5s_4] |
| Sequence 47 | AACTGAAGAGTTTGATCATGGCTCAGATTGAACGC | 1 | 1 | Acinetobacter haemolyticus ATCC 19194 genomic scaffold SCAFFOLD7 |
| Sequence 48 | AAGACGGAAAGACCCCGTGAACC | 1 | 1 | Cardiobacterium hominis ATCC 15826 genomic scaffold SCAFFOLD2 |
| Sequence 49 | AAGACTCGTGAAGCTGACCAGTACTAATAGGCCGA | 1 | 1 | Micrococcus luteus SK58 ctg1119142780300 |
| Sequence 50 | AAGTTTATTAGAGGTAGAGCTACTGATTGGATGCG | 1 | 1 | Chryseobacterium gleum ATCC 35910 Contig281 |
| Sequence 51 | AATCCTGTCTGAACATGGGGGGACCACCCTCCAAG | 1 | 1 | 23S ribosomal RNA\| [locus_tag=Swit_R0028] |
| Sequence 52 | ACGGATCTGTGTAGGATAGGTGGGAGGCTTTGAAG | 1 | 1 | 23S ribosomal RNA\| [locus_tag=DelCs14_R0095] |
| Sequence 53 | ACTGTAGGGGGTAGAGCACTGTTTCGGCTAGGGGG | 1 | 1 | 23S ribosomal RNA\| [locus_tag=PSEBR_23r4] |
| Sequence 54 | AGAAGGTCTTCGGATTGTAAAGC | 1 | 2 | Megasphaera sp. UPII 199-6 contig00034 |
| Sequence 55 | AGCGGAACGCTCTGGAAAGTGC | 1 | 2 | 23S ribosomal RNA\| [locus_tag=PSEBR_23r4] |
| Sequence 56 | AGCGTTAATCGGAATTACTGGGC | 1 | 1 | Providencia rustigianii DSM 4541 genomic scaffold Scfld6 |
| Sequence 57 | AGCTCGTGTCGTGAGATGTCGG | 1 | 1 | 16S ribosomal RNA\| [locus_tag=Psta_R0021] |
| Sequence 58 | AGGCGCTTGAGAGAACTCTGGAGA | 1 | 2 | 23S ribosomal RNA\| [locus_tag=Lcho_R0016] |
| Sequence 59 | AGGGATCGTGTGGGAGAGTAAGACGCTGCCGGAAT | 1 | 1 | Propionibacterium acnes HL002PA1 genomic scaffold Scfld0 |
| Sequence 60 | AGGGGGTAGAGCACTGTTTCGGC | 1 | 2 | 23S ribosomal RNA\| [locus_tag=PSEBR_23r4] |
| Sequence 61 | AGTTGGGCTTACGCCCTGTGAGACTAGAACGTTGC | 1 | 1 | Gemella haemolysans ATCC 10379 ctg1119035638545 |
| Sequence 62 | ATCGTGACGATAGTGCAATGGC | 1 | 1 | 23S ribosomal RNA\| [locus_tag=Daci_R0090] |
| Sequence 63 | ATTCTCAGGCGCTTGAGAGAACTCTGGAGAAGGAA | 1 | 1 | 23S ribosomal RNA\| [locus_tag=Lcho_R0016] |
| Sequence 64 | ATTTGCTTGTGTAGGATAGGTGGGAGGCTTTGAAG | 1 | 19 | 23S ribosomal RNA\| [locus_tag=PSEBR_23r4] |
| Sequence 65 | ATTTTGGACAATGGGCGCAAGC | 1 | 1 | Neisseria flavescens SK114 ctg1118407793883 |
| Sequence 66 | CATCGTGACGATAGTGCAATGGC | 1 | 2 | 23S ribosomal RNA\| [locus_tag=Daci_R0090] |
| Sequence 67 | CGAGGGATCGTGTGGGAGAGTAAGACGCTGCCGGA | 1 | 2 | Propionibacterium acnes HL002PA1 genomic scaffold Scfld0 |
| Sequence 68 | CGCGGGGTGGAGCAGTCTGGTAGCTCGTTGGGCTC | 1 | 1 | Met tRNA\| [locus_tag=Mpe_AR0064] |
| Sequence 69 | CGGAGTGTGGCTCAGCCCGGTA | 1 | 1 | Bradyrhizobium japonicum USDA 110 chromosome |
| Sequence 70 | CGGATCTGTGTAGGATAGGTGGGAGGCTTTGAAGT | 1 | 4 | 23S ribosomal RNA\| [locus_tag=DelCs14_R0095] |
| Sequence 71 | CGGATTGTAAAGCACTTTAAGTTGGGAGGAAGGGC | 1 | 1 | 16S ribosomal RNA\| [gene=rrsA] [locus_tag=PSPA7_0811] |
| Sequence 72 | CGGCCTGGTGGTTCTAGCGAAGC | 1 | 1 | Bradyrhizobium sp. S23321 5S ribosomal RNA |
| Sequence 73 | CGGGAAGTAGCTCAGCTTGGTAGA | 1 | 1 | Streptococcus sanguinis SK678 genomic scaffold SCAFFOLD1 |
| Sequence 74 | CGTGGAGCTGACGAATACTAATCGATCGAAGACTT | 1 | 2 | Staphylococcus epidermidis SK135 ctg1119966624826 |
| Sequence 75 | CGTTATCCGGATCGGGGACAGTGC | 1 | 1 | Achromobacter piechaudii ATCC 43553 genomic scaffold SCAFFOLD1 |
| Sequence 76 | CTGTGTAGGATAGGTGGGAGGC | 1 | 1 | Roseomonas cervicalis ATCC 49957 genomic scaffold SCAFFOLD3 |
| Sequence 77 | CTTAAGTGGCTTTGAGATTAGCGGAACGCTCTGGA | 1 | 1 | 23S ribosomal RNA\| [locus_tag=Pfl01_R15] |
| Sequence 78 | GAACGGATCTGTGTAGGATAGGTGGGAGGCTTTGA | 1 | 1 | 23S ribosomal RNA\| [locus_tag=DelCs14_R0095] |
| Sequence 79 | GAACTGTAGAGTTTGATCCTGGCT | 1 | 1 | Alicycliphilus denitrificans BC chromosome |
| Sequence 80 | GAGTTTGATCCTGGCTCAGAGCGAACGCTGGCGGC | 1 | 2 | 16S ribosomal RNA\| [locus_tag=Mext_R0034] |
| Sequence 81 | GAGTTTGATCCTGGCTCAGATTGAACGCTGGCGGC | 1 | 1 | Neisseria polysaccharea ATCC 43768 N_polysaccharea-1.0.1_Cont168.1 |
| Sequence 82 | GATAGGTGGGAGGCTTTGAAGCG | 1 | 1 | Providencia rustigianii DSM 4541 genomic scaffold Scfld6 |
| Sequence 83 | GCCGAAAGGCGTAGTCGATG | 1 | 1 | Enterobacter cancerogenus ATCC 35316 genomic scaffold Scfld3 |
| Sequence 84 | GCGTAGAGATCTGGAGGAATAC | 1 | 1 | Escherichia coli 83972 genomic scaffold SCAFFOLD11 |
| Sequence 85 | GCGTTAATCGGAATTACTGGG | 1 | 1 | Providencia rustigianii DSM 4541 genomic scaffold Scfld6 |
| Sequence 86 | GCTAGTTACGGTCGGACATCGT | 1 | 1 | 23S ribosomal RNA\| [locus_tag=Daci_R0090] |
| Sequence 87 | GCTCAGACCGGTCGGAAATCGG | 1 | 1 | 23S ribosomal RNA\| [locus_tag=PSEBR_23r4] |
| Sequence 88 | GGATCTGTGTAGGATAGGTGGGAGGCTTTGAAGTG | 1 | 1 | 23S ribosomal RNA\| [locus_tag=DelCs14_R0095] |
| Sequence 89 | GGCCTGAGAGGGCGACCGGCCACACTGGGACTGAG | 1 | 1 | Brevibacterium mcbrellneri ATCC 49030 contig00111 |
| Sequence 90 | GTACCGGAGTGGGTGAGAGTCCTGTAACTGTAAGC | 1 | 1 | Propionibacterium acnes HL002PA1 genomic scaffold Scfld0 |
| Sequence 91 | GTGAAACTCAAAGGAATTGACGGGGGCCCGCACAA | 1 | 1 | Prevotella veroralis F0319 P_veroralis-1.0.1_Cont1.2 |
| Sequence 92 | GTGAAATAGATCCTGAAACCGCATGCATACAAAAA | 1 | 1 | 23S ribosomal RNA\| [locus_tag=Lcho_R0016] |
| Sequence 93 | GTGGGGAATTTTGGACAATGGGCGAAAGCCTGATC | 1 | 1 | Ralstonia sp. 5_7_47FAA genomic scaffold supercont1.15 |
| Sequence 94 | GTTAATCGGAATTACTGGGCGTAAAGCGTGCGCAG | 1 | 1 | Achromobacter piechaudii ATCC 43553 contig00178 |
| Sequence 95 | TGAACGGATCTGTGTAGGATAGGTGGGAGGCTTTG | 1 | 2 | 23S ribosomal RNA\| [locus_tag=DelCs14_R0095] |
| Sequence 96 | TGAGACTGTGGGGGATAAGCTCCA | 1 | 1 | Mobiluncus curtisii ATCC 51333 genomic scaffold SCAFFOLD2 |
| Sequence 97 | TGGAGCATGTGGTTTAATTCGAAGCAACGCGAAGA | 1 | 1 | Enterococcus faecalis S613 E_faecalisS613-1.0_Cont116.1 |
| Sequence 98 | TGGTGGGAACTCTAAGGAGACTGC | 1 | 1 | Salinicola sp. PM-10 16S ribosomal RNA gene |
| Sequence 99 | TGTGAAAGTAGGACATCGTCAGGC | 1 | 1 | 5S ribosomal RNA\| [gene=rrfA] [locus_tag=Rta_05449] |
| Sequence 100 | TGTGGAGCTGACTGGTACTAATAGACCGAGGACTT | 1 | 1 | Propionibacterium acnes HL002PA1 genomic scaffold Scfld0 |
| Sequence 101 | TTAGCGGAACGCTCTGGAAAGTGC | 1 | 2 | 23S ribosomal RNA\| [locus_tag=PSEBR_23r4] |
| Sequence 102 | TTGCCTCTTGGGGTTGTAGGACAC | 1 | 1 | 23S ribosomal RNA\| [locus_tag=Aflv_R004] |
| Sequence 103 | TTTGAACGGATCTGTGTAGGATAGGTGGGAGGCTT | 1 | 4 | 23S ribosomal RNA\| [locus_tag=DelCs14_R0095] |
| Sequence 104 | GAATCTGAGGGTCGGACGTTCGAATCGTTCCGGGC | 8 | 1 | Bradyrhizobium sp. BTAi1 chromosome |
| Sequence 105 | TCTGAATATGGGGGGACCATCCTCCAAGGCTAAAT | 8 | 1 | Providencia rustigianii DSM 4541 genomic scaffold Scfld6 |
| Sequence 106 | TAAACTGAAGAGTTTGATCCTGGC | 6 | 1 | Achromobacter piechaudii ATCC 43553 contig00178 |
| Sequence 107 | GCCACACTGGGACTGAGACACGGC | 5 | 1 | Enterococcus faecalis S613 E_faecalisS613-1.0_Cont116.1 |
| Sequence 108 | TTAACTGGAGAGTTTGATCCTGGCTCAGAACGAAC | 5 | 1 | 16S ribosomal RNA\| [locus_tag=sce_rRNA_16_2] |
| Sequence 109 | CCACACTGGGACTGAGACACGGC | 4 | 2 | Enterococcus faecalis S613 E_faecalisS613-1.0_Cont116.1 |
| Sequence 110 | CGGGATGTAGCGCAGTTTGGTAGC | 4 | 1 | Mitsuokella multacida DSM 20544 M_multacida-1.0.1_Cont1.2 |
| Sequence 111 | CTCAGATTGAACGCTGGCGGC | 4 | 1 | Cardiobacterium hominis ATCC 15826 genomic scaffold SCAFFOLD2 |
| Sequence 112 | GGGATTGTAGTTCAATTGGTTAGA | 4 | 15 | Asp tRNA\| [locus_tag=MAE_t001] |
| Sequence 113 | CGAACTGTAGAGTTTGATCCTGGCTCAGATTGAAC | 26 | 1 | Alicycliphilus denitrificans K601 chromosome |
| Sequence 114 | GAACTGAAGAGTTTGATCATGGCTCAGATTGAACG | 12 | 17 | Methylomicrobium alcaliphilum 16S rRNA |
